# Isoforms of the p53 Family and Gastric Cancer: A Ménage à Trois for an Unfinished Affair

**DOI:** 10.3390/cancers13040916

**Published:** 2021-02-22

**Authors:** Anais Blanchet, Agathe Bourgmayer, Jean-Emmanuel Kurtz, Georg Mellitzer, Christian Gaiddon

**Affiliations:** 1 Laboratory “Streinth” (STress REsponse and INnovative Therapies in Cancer), Inserm UMR_S 1113, IRFAC, ITI InnoVec, Université de Strasbourg, 67200 Strasbourg, France; anais.blanchet2@etu.unistra.fr (A.B.); a.bourgmayer@icans.eu (A.B.); je.kurtz@icans.eu (J.-E.K.); mellitzer@unistra.fr (G.M.); 2 ICANS (Institut du Cancer Strasbourg Europe), 67200 Strasbourg, France

**Keywords:** p53 family, isoforms, gastric cancer, biomarker, personalized therapy, chemotherapy, immunotherapy, metastasis, cell death, tumor ecosystem

## Abstract

**Simple Summary:**

The p53 family is a complex family of transcription factors with different cellular functions that are involved in several physiological processes. A massive amount of data has been accumulated on their critical role in the tumorigenesis and the aggressiveness of cancers of different origins. If common features are observed, there are numerous specificities that may reflect particularities of the tissues from which the cancers originated. In this regard, gastric cancer tumorigenesis is rather remarkable, as it is induced by bacterial and viral infections, various chemical carcinogens, and familial genetic alterations, which provide an example of the variety of molecular mechanisms responsible for cell transformation and how they impact the p53 family. This review summarizes the knowledge gathered from over 40 years of research on the role of the p53 family in gastric cancer, which still displays one of the most elevated mortality rates amongst all types of cancers.

**Abstract:**

Gastric cancer is one of the most aggressive cancers, with a median survival of 12 months. This illustrates its complexity and the lack of therapeutic options, such as personalized therapy, because predictive markers do not exist. Thus, gastric cancer remains mostly treated with cytotoxic chemotherapies. In addition, less than 20% of patients respond to immunotherapy. *TP53* mutations are particularly frequent in gastric cancer (±50% and up to 70% in metastatic) and are considered an early event in the tumorigenic process. Alterations in the expression of other members of the p53 family, i.e., p63 and p73, have also been described. In this context, the role of the members of the p53 family and their isoforms have been investigated over the years, resulting in conflicting data. For instance, whether mutations of *TP53* or the dysregulation of its homologs may represent biomarkers for aggressivity or response to therapy still remains a matter of debate. This uncertainty illustrates the lack of information on the molecular pathways involving the p53 family in gastric cancer. In this review, we summarize and discuss the most relevant molecular and clinical data on the role of the p53 family in gastric cancer and enumerate potential therapeutic innovative strategies.

## 1. p53 and the Original Sin

The p53 protein was originally discovered in 1979 as a cellular binding partner of the Large T antigen of the Simian Virus 40 [1,2]. It was soon shown to be a phosphorylated protein [3,4] more highly expressed in cancer cells than in normal cells [5], characterized by a low stability that was controlled by posttranslational processes [6], and able to bind DNA [7]. At the cellular level, the p53 protein was found to be capable of inhibiting cell growth [8]. Later on, the *TP53* gene that encodes the p53 protein was established as a tumor suppressor gene and was found to be the most commonly mutated gene in cancers, with approximately 50% penetrance [9,10]. About 20 years later, two paralogs of *TP53*, *TP63*, and *TP73* were discovered. The successive investigations performed on p53 mutations in gastric cancer (GC) over the years is an archetype of the research done to combat cancer that have been paved with successes, defeats, and confrontative theories. 

## 2. Gastric Cancer, a Disregarded Health Issue in Most of the Western Countries

Gastric cancer (GC) is the fifth most common cancer in the world, mostly affecting men (sex ratio of 2.5). Its mortality remains strikingly high compared to other types of cancers (third in the world and 800,000 deaths in 2018), with a significant geographic disparity between Asia and Europe [11,12]. For instance, in Europe, the five-year survival rate is less than 25% in all stages combined, and the median survival is about 11 months. The GC incidence is higher in Asia (32 per 100,000 among males) than in Western countries (5.6 per 100,000), probably due to food habits and genetic differences. Hence, research in GC is more developed in Asia in contrast to most Western countries where less effort and resources are invested in research and treatment, especially by the pharmaceutical companies. For instance, in Japan, 70% of localized gastric cancers are cured in the setting of early diagnosis owing to a systematic screening policy. Risk factors include bacterial (*Helicobacter pylori*) and viral (Epstein-Barr virus, EBV) agents, as well as rare hereditary mutations in the *CDH1* gene or in Mismatch Repair genes involved in the hereditary nonpolyposis colorectal cancer (HNPCC) or Lynch syndrome [13]. In addition, lifestyle factors like food preferences (e.g., salty or smoked foods), tobacco, and alcohol are important risk factors accounting for the presence of synchronous cancers in the stomach and oral cavity [14]. Inversely, the consumption of vegetables (e.g., carrots, lettuce) reduces the risk of GC [15,16].

**Figure 1:**
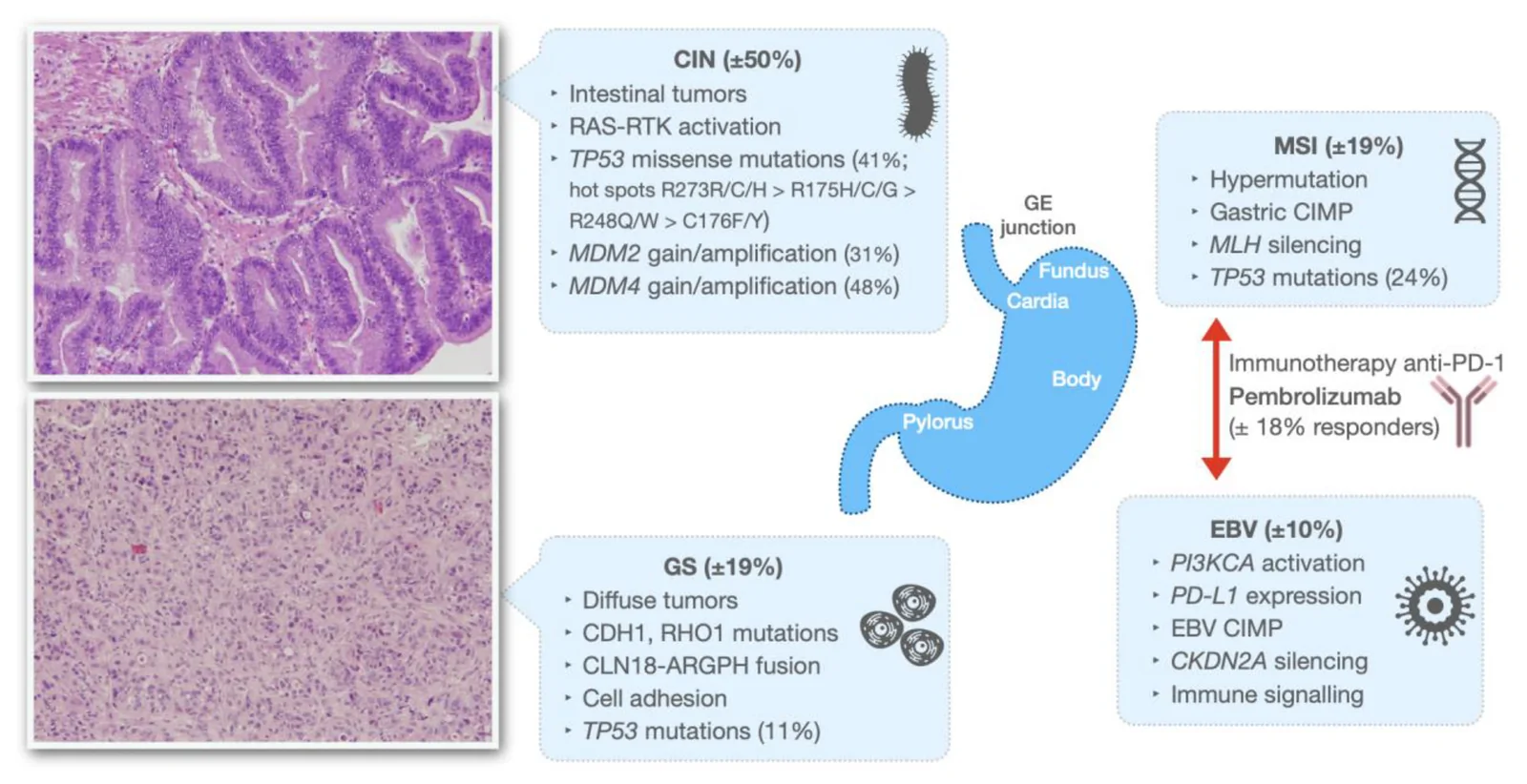
Histological and molecular subgroups of gastric cancer. Gastric cancer has two major histological subtypes: the intestinal subtype (upper left) that presents differentiated cancer cell structures in glands, and the diffuse subtype (lower left) that displays undifferentiated cancer cells dispersed within the stroma. There are also four molecular subgroups defined by the TCGA: the Chromosome instable (CIN), the Genetic Stable (GS), the Microsatellites Instable (MSI), and the Epstein–Barr Virus (EBV) subgroups. Each are characterized by specific deregulated mechanisms. Of note is the two molecular subgroups that tend to respond the best to immunotherapy, the MSI and the EBV subgroups.

There are two major histological GC subtypes representing 90% of cases: intestinal and diffuse (e.g., signet ring cell type) cancers. The intestinal subtype presents a cellular organization of the cancer cells similar to intestinal epithelial glands (Figure 1). The diffuse subtype is characterized by the dispersion of cancer cells within the stroma [17]. More recently, a molecular classification has been established identifying four molecular groups. [18]. The most important subgroup, matching mostly the intestinal subtype, is “Chromosomal Instability” (CIN, 49%), characterized by *TP53* mutations and activation of the RTK-RAS pathway. The “Epstein-Barr virus” subgroup (EBV, 9%) is associated with DNA hypermethylation, frequent *PIK3CA* mutation, *CDKN2A* silencing, and overexpression of PD-L1/2. The “Microsatellite Instable” (MSI, 22%) subgroup has *MLH1* silencing and a hypermutation phenotype, making these last two subgroups candidates for immunotherapy with immune checkpoints inhibitors [19]. Finally, the “Genomic Stable” subgroup (GS, 20%) corresponds to *CDH1* and *RHOA* mutations and matches the diffuse GC subgroup. *TP53* mutations are also found in the MSI and GS subgroups.

Surgery with perioperative platinum-based chemotherapy (e.g., oxaliplatin) is the gold standard treatment for localized GC, but surgical morbidity is reported to be as high as 39% [20]. The low five-year survival rate (25%) in Europe highlights an unmet medical need, which is due to inherent or acquired resistance mechanisms, like *EGFR* overexpression [21] or the aberrant activation of various circulating microRNAs (miRNAs) [22]. So far, the molecular classification has not led to the introduction of new routine treatments, and even immunotherapy strategies have led to only limited advances with less than 20% of responders. One of the challenges is to identify the patients that have a better chance of responding to immunotherapy. 

The unmet need for truly efficient treatments in advanced GC illustrates the unique complexity of this cancer. Indeed, despite the classifications and molecular data, we are still missing some crucial information required to develop new treatments that would improve the patients’ management for survival benefit. This requires going beyond the existing classifications and refinement of our understanding of the molecular mechanisms that are often oversimplified and/or overlooked despite their extreme complexity. For instance, pan-genomic analyses do not include data on the relative expression of isoforms generated by alternative splicing or promoters or on protein expression levels that can be controlled through complex post-transcriptional modifications impacting protein stability, subcellular localization, and activity. 

**Figure 2:**
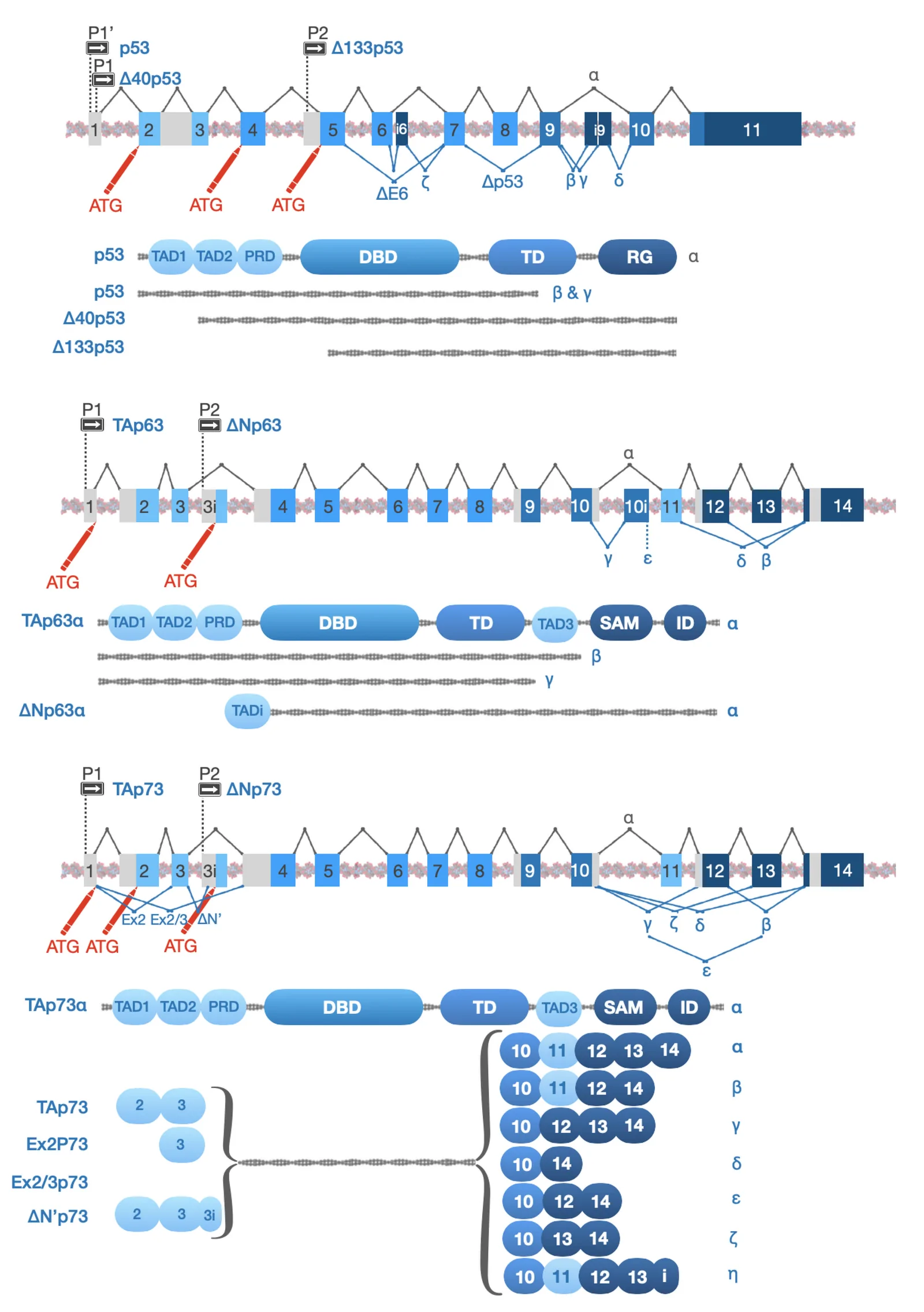
The isoforms of the p53 family. The p53 family has 3 genes: *TP53*, *TP63*, and *TP73*, that express multiple isoforms via alternative promoters (P1, P1′, and P2), generating full-length proteins with N-terminus transactivation domains (e.g., TA) or without (e.g., ∆N) and alternative splicing generating variations in the C-terminus (e.g., α, β, and γ). The encoded proteins contain transactivation domains (TAD1, 2, and 3); a proline-rich region (PRD); a DNA-binding domain (DBD), tetramerization domain (TD), sterile α domain (SAM), and an inhibitor domain (ID).

A good example of this is the complex p53 family of transcription factors that is present at the center of a dense interdigitated network of signaling pathways. The p53 family is composed of three genes: *TP53* (p53), *TP63* (p63), and *TP73* (p73), and each generates multiple isoforms via alternative promoters and splicing (Figure 2). Importantly, the function of these isoforms can be opposing, as they generate isoforms with a N-terminus transactivation domain (TA isoforms) or without it (∆N isoforms). The function of these genes in cancers have been investigated over the years and were found to be critical for tumorigenesis, response to treatment, and metastasis [23]. In this comprehensive review that compiles about 400 articles out of 2600 published on the p53 family in GC, we are focusing on their expression and function in GC to highlight what has been clearly established but, also, what remains to be understood for a successful transfer to patient care.

## 3. *TP53*, a Guardian Against Gastric Cancer?

### 3.1. Incidence of *TP53* Mutations in Gastric Cancer

**Figure 3:**
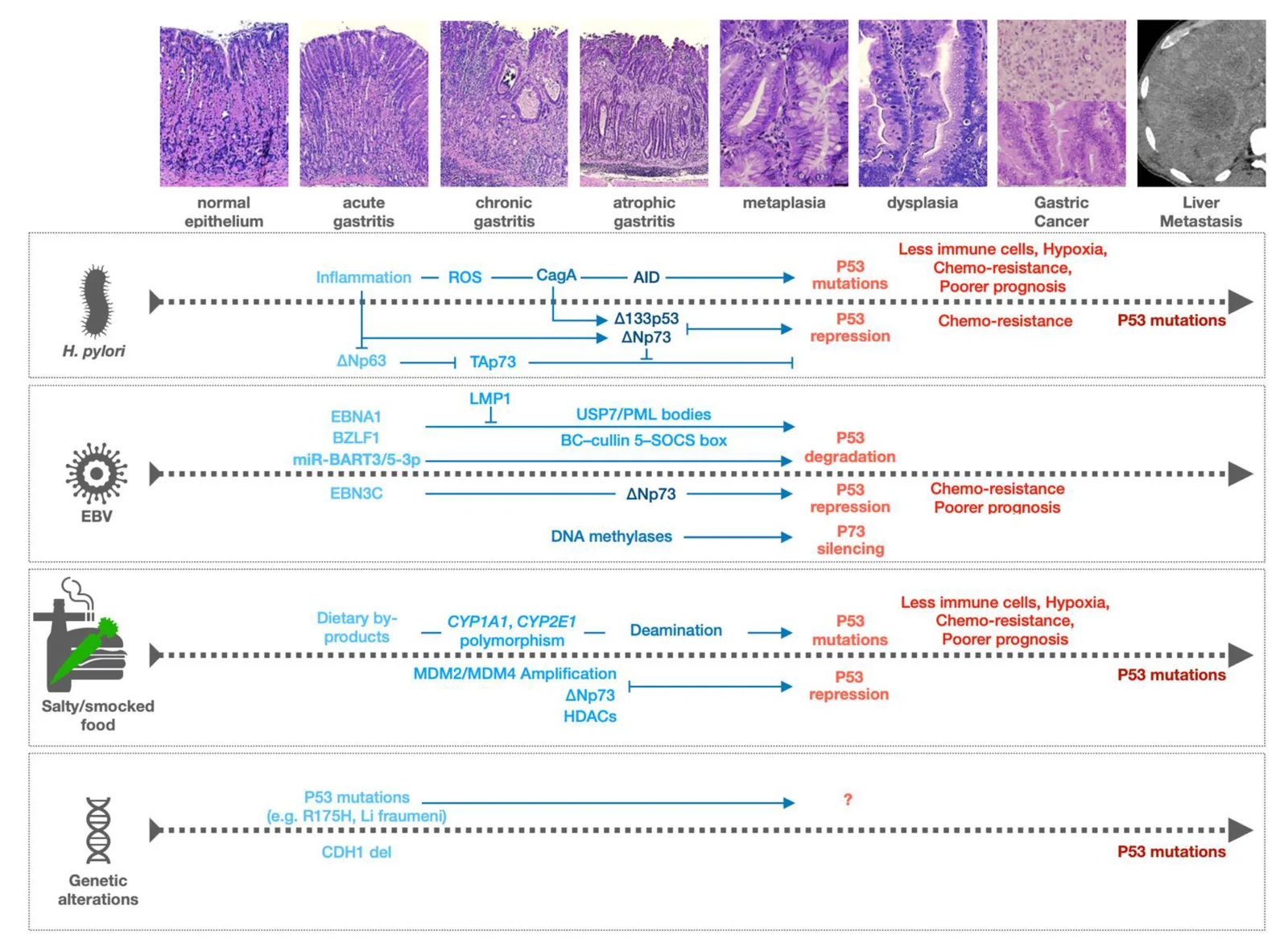
Deregulation affecting the isoforms of the p53 family in gastric cancer. Mutations in *TP53* or alterations in the expression of *TP53*, *TP63,* and *TP73* isoforms are observed during gastric tumorigenesis, evolving from a normal stomach epithelium toward adenocarcinomas via gastritis, metaplasia, and dysplasia. Images taken 20X.

Drs. Sano and Tahara were the first to report a loss of heterozygosity (LOH) in the *TP53* locus, 17p13.1, in 68% of gastric tumors (*n* = 48) [24]. The *TP53* LOH frequency was higher in well-differentiated cancers and in advanced cancers (up to 70% in metastatic cancers). Simultaneously, Drs. Tamura and Hirohashi detected *TP53* mutations in exons 4–8 (codons deletions 137–139, V173M, I251S, insertion *CTCA* 252–253) in 64% (*n* = 24) of aneuploid tumors but none in diploid tumors [25]. They suggested that *TP53* mutations were occurring at a late stage of the carcinogenesis, as subsequently supported by other studies (e.g., codons V216L and R248W) [26,27]. As seen in other types of cancers, the mutations were mostly centered in the most conserved region of the p53 protein, the DNA-binding domain [27]. The accumulation of mutated p53 proteins in malignant GC cells was confirmed by Dr. Lane using monoclonal antibodies [28]. These mutations were also observed in GC cell lines that are still widely used nowadays, such as KATOIII (deleted for *TP53*), NUGC-3 (p53 Y220C), MKN28 (p53 I251L), or MKN1 (p53 V143A) [29]. However, a year later, Drs. Yokosaki and Tahara showed that, even in primary tumors, *TP53* was mutated (codons G117A, C141T, W158C, M160K, G226A, D228T, G245S, I254T, and V272E) in 56% of the samples, including early-stage cancers I and II [30] (Figure 3). It turned out that *TP53* mutations were homogeneously present within the tumors, even at the early stages [31,32,33], including stomach precancer states such as incomplete intestinal metaplasia [34] and gastritis induced by *H. pylori* infection [35]. Altogether, these observations indicate that *TP53* mutations occur before clonal expansion and, therefore, are likely a founding event in GC carcinogenesis and critical for the transformation of stomach epithelial cells. 

In addition, familial mutations in *TP53*, like at aa 175 for Li-Fraumeni or the atypical truncation E287X [36], lead to GC (±5% incidence) but less frequently to colon cancer [37,38,39]. Hence, the carcinogenesis processes seem to be different between GC and colon cancer, where alterations of *APC* are more prominent than of *TP53* [40]. However, the low penetrance of GC in Li-Fraumeni patients, which is higher in families of Asian origin than in the Western population, indicates the existence of genetic/environmental modulators and raises interesting questions about complementary/compensatory mechanisms [41].

The prominent role of p53 in GC raises the questions of both the origin of these events and their influence on gastric epithelial cell transformations towards the development of particular GC subtypes.

### 3.2. Association of p53 Mutations with Specific Types of Gastric Cancers

The pattern of *TP53* mutations in GC shows complexity, as they are frequently present at an early stage of the intestinal subtype (41% *n* = 130) and rarely in the diffuse subtype (4%), but the mutation frequency increases significantly up to 33% in the late stage of the diffuse subtype [42]. This suggested a differential role of p53 in both types of cancers, which was further supported by several studies [43,44]. *TP53* mutations were found more likely to be associated with *H. pylori* infection, a major cause for the development of GC of the intestinal subtype [35,45]. In addition, the involvement of *TP53* mutations in the intestinal type is well-demonstrated by mouse genetic experiments in which precancerous lesions (polyps) and carcinomas following the exogenous overexpression of the intestinal differentiation transcription factor CDX2 are favored by the inactivation of *tp53* (50 weeks, 50% penetrance vs. 80–100 weeks) [46]. The inactivation of *TP53* might still be needed for the development of the diffuse type, since the amplification of the major p53 inhibitor, *MDM2*, was described to be more frequently found in this subtype [47]. However, a detailed analysis of the TCGA (The Cancer Genome Atlas) data do not support this early observation (Figure 1, personal data). The differential role of *TP53* mutations is also observed with regards to the location of the tumor. For instance, tumors of the cardia seem to have more frequent mutations (54%) than tumors of the antrum (25%) [48]. In addition, *TP53* mutations are also present in the rare gastric carcinomas “small cell carcinoma” [49]. 

Pan-genomic analyses of GC defined the molecular subgroups and indicated that *TP53* mutations are mostly associated with the CIN molecular subgroup that represents about 49% of GC [18] (Figure 1). This observation is in agreement with previous observation, as the CIN molecular subgroup mostly corresponds to the intestinal subtype often induced by *H. pylori* infection. However, a careful analysis of the TCGA shows that *TP53* mutations are also present in the MSI (24% of *TP53* missense mutations) and the GS subgroups (11% of missense mutations). In addition, truncating mutations are present in 28%, 9%, and 2% of the CIN, MSI, and GS subgroups, respectively (personal data). 

**Figure 4:**
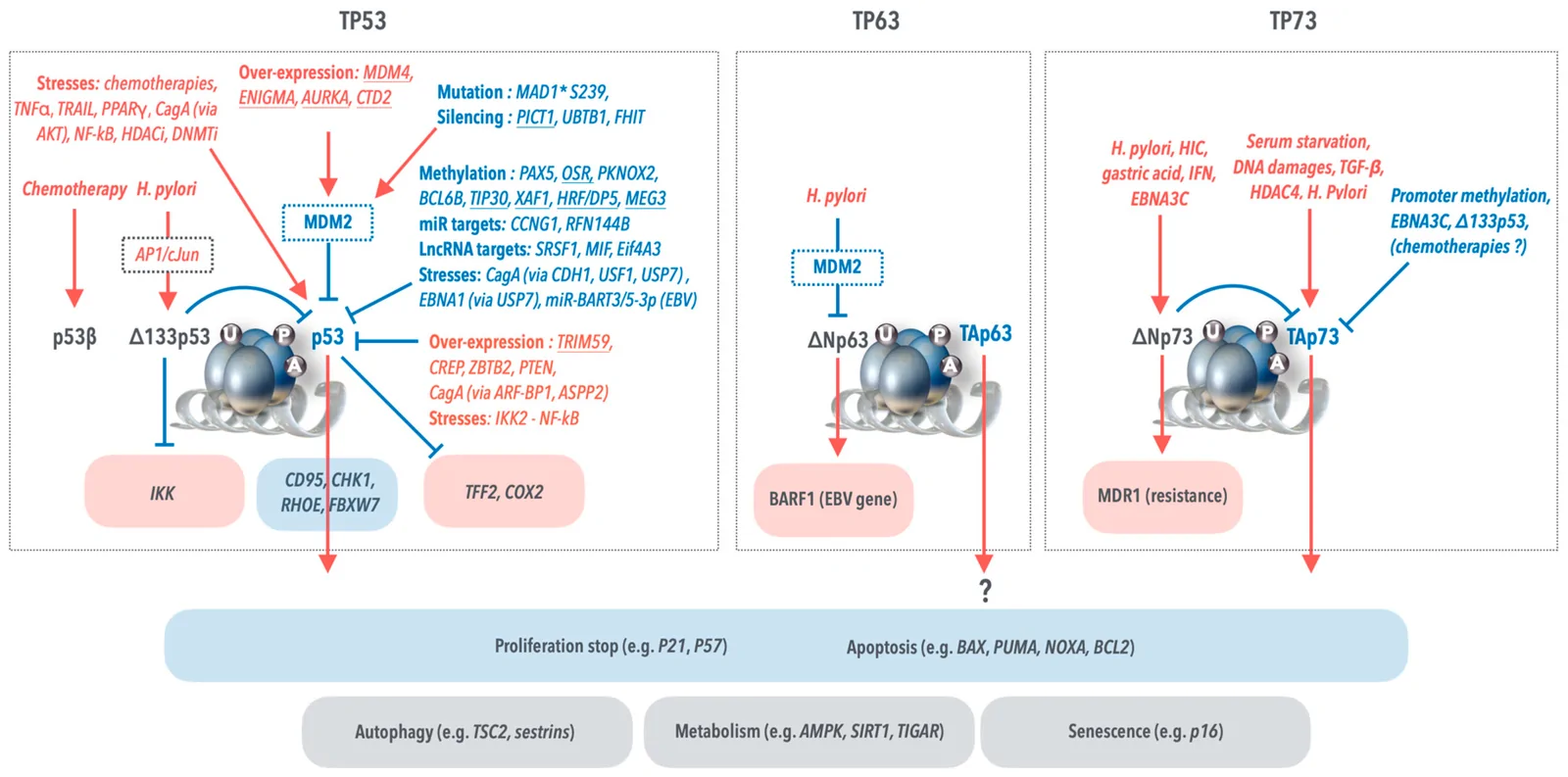
Signals and mechanisms affecting the expression and activity of the isoforms of the p53 family in gastric cancer. Activators are indicated in red and repressors in blue. Underlines indicate existing clinically relevant data.

In addition to pan-genomic analyses, *TP53* mutations have been correlated with precise molecular alterations of genes that may or may not be related to p53. For instance, *TP53* mutations are more frequently (81%) found in tumors overexpressing *EGFR/TGFα* (26%) [50] or *CHK1/2* [51]. In contrast, the epigenetic silencing of *XAF1* by promoter methylation, which interacts with the *DIABLO* proapoptotic protein, is exclusive of *TP53* mutations, suggesting that they participate in the same pathway [52] (Figure 4). Indeed, *XAF1* interferes in a complex way with *p53* function [53], and *p53* represses the expression of *XAF1* at the transcriptional level in GC cells [54,55]. Similar observations were made with *HRK/DP5* [56]. More generally, pan-genomic analyses established that *TP53* mutations are exclusive with the methylation of several tumor suppressor genes, such as *PGP9.5*, *NMDAR2B*, or *CCNA1* [57].

### 3.3. Causes for *TP53* Mutations in Gastric Cancer

It is proposed that *TP53* mutations are caused by dietary carcinogens that induce DNA deamination [32]. This is supported by mouse experiments showing that the food carcinogen MelQ (2-Amino-3,4-dimethylimidazo [4,5-f]quinoline) and the alkylating agent N-methyl-N-nitrosourea (MNU) can induce GC with p53 mutations (aa 171 or 113) [58,59,60]. In addition, the transformation ability of MNU is higher (incidence and aggressivity with invading mucosa) in mice with inactivated *tp53* [61,62]. Moreover, it was observed that patients drinking alcohol with polymorphisms in detoxifying enzymes (ex., *CYP1A1* and *CYP2E1*) have more mutations in *TP53* [63] (Figure 3), which further supports the correlation between carcinogens and *TP53* mutations. 

Another source of *TP53* mutations is *H. pylori* infection, which highly correlates with the infected gastric mucosa of humans and Japanese monkeys [64]. the infection by *H. pylori* causes genomic DNA oxidation in various genes due to inflammation and the production of radical oxidized species (ROS), leading to mutations in the *TP53* gene even at the gastritis stage [35,65,66] (Figure 3). Additional mechanisms have been proposed, such as the cytidine deamination of the *TP53* DNA sequence by the induction of *AID* (Activation-Induced cytidine Deaminase) following NF-kB (Nuclear Factor kappa B) activation by the *H. pylori* protein *CagA* [67,68]. Indeed, accumulated mutations were predominantly C:G > T:A transitions in GpCpX motifs—a marker of cytidine deamination by *AID*. In addition, the expression of *AID* in mice induces *TP53* mutation [66]. Finally, *TP53* mutations following *H. pylori* infection also involved *CHAC1* (Cation transport regulator 1) that has γ-glutamylcyclotransferase activity to degrade glutathione, an antioxidant [69]. 

The role of food carcinogens, as well as the specificity of the *H. pylori* strain, may explain some observations made towards ethnical differences. For instance, the Europeans show more G:C to A:T transitions (90% vs. 48%) and the Oriental population more A:T to G:C transitions (22% vs. 2%) [70]. Similarly, more frequent *TP53* mutations are found in the GC of African Americans compared to other ethnic groups [71]. 

### 3.4. *TP53* Polymorphism and Gastric Cancer

*TP53* presents polymorphisms in exon 4 at codon 36 and 72 that impact the prognosis of different cancers [72]. The most common polymorphism is at codon 72 (*pro* vs. *arg*) and differs between populations. The impact of this polymorphism in GC is still debated in the literature. Several studies or meta-analyses suggest that there is no difference in polymorphism representation in GC patients compared to the general population [73]. However, other analyses suggest that the impact of the polymorphism may be dependent on specific subtypes (lower frequency of *Pro/Pro* in poorly differentiated cancer) and the ethnicity (in Asians: less cancer with *Arg/Arg* compared to the general population, *Pro/Pro* more present in the cardia subtype, and *Arg/Arg* more present in advanced cancers) [74]. The more common *Arg/Arg* codon 72 polymorphism in Caucasians correlates with a tendency for cardia GC, whereas the more common *Pro* allele in African Americans correlates with a tendency for antral GC [72,75]. In addition, the *Arg/Arg* polymorphism favors the survival of patients with non-cardia cancer [75], although it increases slightly the risk (adjusted odds ratio (OR) = 1.67, 95% confidence interval (CI) = 1.00–2.77) of non-cardia GC, especially in alcoholics [76]. Furthermore, the *Pro* polymorphism could also favor the development of GC from gastritis [77]. In addition, the *Arg/Pro* and *Pro/Pro* genotypes significantly correlate with a lower response rate to chemotherapy when compared to the *Arg/Arg* genotype (35.7 vs. 66.7%, *p*-value 0.019) [78,79], but the *Arg/Arg* polymorphism is of negative predictive value for 5-fluorouracil (FU) -based therapy [80]. Altogether, it seems that aa 72 polymorphisms may have a predictive significance in GC, but their exact roles are complex. 

Based on the fact that *TP53* mutations occur early during the gastric epithelial cell transformation and that p53 is an important factor in the response to DNA damages, it seems relevant to consider that the p53 status (mutation or expression level) might be used as a biomarker for personalized therapy.

### 3.5. Are *TP53* Mutations Biomarker for Personalized Therapy in Gastric Cancer?

Two studies initially showed that the mutational status of *TP53* was an independent biomarker for a reduced five-year survival (9% vs. 42%, *n* = 116 patients) [81] and for a poor response to *cisplatin* associated with a low *P21* expression and reduced apoptosis (heterozygote for R162P, R175H, P177S, A178D, R213P, Y220C, R273C, and R282L, *n* = 11 patients) [82]. However, soon after, the value of p53 mutations as a prognosis marker was debated between studies supporting the early results (ex., [83]), including by using circulating DNA (ctDNA) to detect *TP53* mutations [84], and others with the opposite conclusion (ex., [85]). These discrepancies may reflect the small size and/or the heterogeneity (grades, subtypes, ages, etc.) of the investigated cohorts. However, a further meta-analysis clearly indicated then that *TP53* mutations should be considered as a predictive marker for the response to chemotherapy [86]. This mutational status of *TP53* as a biomarker for a poor prognosis is in agreement with additional observations that GC patients with *TP53* mutations tend to develop more aggressive tumors characterized by frequent liver metastasis (86% vs. 40%) [87] and the presence of a hypoxic response [88] (Figure 3). 

In order to increase the specificity and perform personalized therapy using the *TP53* status as a biomarker, combined analyses of different genes associated with *TP53* mutations have been tested. For instance, the combined mutation of *TP53* with *BAX* [89] or *NRXN1* expression [90] drastically worsened the prognosis for GC patients. Inversely, *TP53* mutations associated with an elevated expression of the oncogenic YAP protein tend, surprisingly, to favor GC patient survival [91]. Although the molecular mechanisms explaining this unexpected observation remain unclear, one may speculate that the pro-proliferative activity of YAP and mutant p53 facilitates the activity of cytotoxic drugs. 

More recently, pan-genomic analyses on large cohorts have shown that *TP53* mutations have an impact on the prognosis in specific molecular subgroups. For instance, *TP53* mutations in tumors with a MSS (microsatellite stable) status negatively impact the patients’ prognosis [92], and the association of p53 mutants with the CIMP (CpG island methylated phenotype) characteristic shows a higher prognosis quality [93]. Similarly, *TP53* mutations are significantly associated with a decrease in the overall survival in patients with diffuse or stage III or IV tumors [94].

A better understanding of the molecular impact of each type of mutation on p53 activity has led to studies querying whether these mutations impact patients’ prognosis differently. Oncogenic *TP53* missense mutations are mostly found in the core domain. Many of these mutations result in protein misfolding and accelerated aggregation. *TP53* mutations also frequently result in the alteration or loss of zinc at the DNA-binding site, which increases the aggregation via nucleation with zinc-bound p53. The top 10 most frequent mutations (hotspots) affect codons of the core residues *R175H*, *Y220C*, *G245S/D/C*, *R248Q/W*, *R249S/C/D*, *R273C/H/S*, and *R282H* and are classified into structural mutants, which alter the p53 core structure, and contact mutants that affect residues at the DNA interface [95]. Both structural and contact mutations result in a loss of function for binding to p53-specific DNA elements. In the last decade, several studies have provided compelling evidence that certain hotspot mutations also confer gain of function (GOF) properties to p53, which support a number of oncogenic activities from cell survival to proliferation [96]. In general, such GOF activities result from newly acquired interactions (“neo-interactions”) of p53 mutants with transcription factors (TFs) or coactivators such as YAP, HSF1, p73, ETS1 and 2, NF-kB subunits, and NRF2, which can be defined as “neo-interactants” [97]. These novel interactions create “neo-TFs”, leading to alterations in the gene expression patterns that inhibit cell death and favor cell growth. In gastric cancers, mutations in the L3 loop; the LSH motif; and mutations considered as hotspots (R175, G245, R248, R273, and R282) correlate more strongly with a poor prognosis [98,99]. Typically, these mutations correlated with a venous invasion in advanced-stage diseases, significantly worse overall survival, and the recurrence of free survival (*p* = 0.001). The negative impact of some of these mutations (e.g., R175H, R248G, and R273H) on cancer cells has been attributed to gain of function properties. Inversely, p53 mutants with residual activity favor survival in males [100]. The impact of these mutations at the molecular level specifically in GC cells has not been well-characterized, except for p53 V143A, which interacts with S100A4 in MKN1 GC cells to stabilize p53 [101]. The silencing of S100A4 reduced the expression of two target genes of p53 V143A: *MYC* and *ID2*.

In summary, there are strong lines of evidence that *TP53* mutations have a negative impact on GC, either by impacting the response to chemotherapy or by favoring the development of metastasis. However, the extent of this impact appears to depend on additional molecular features of the tumors, such as the microsatellite status or mutations in other genes. So far, these potential biomarkers have not been used in the clinic, as no therapeutic alternatives have been or are proposed to treat patients besides chemotherapy. However, in the era of immunotherapy, the recent findings on p53 mutants might point to new therapeutic directions.

Indeed, studies using immunogenics (characterization of the immune landscape using pan-genomics) suggest that *TP53* mutations reduce GC immunogenicity [102]. Strikingly, pan-genomic analyses show that almost all immune gene sets are significantly downregulated in mutated *TP53* vs. wild-type (WT) *TP53* GC. These downregulated immune pathways and cell types in *TP53*-mutated tumors include 15 immune cell types and functions, tumor-infiltrating lymphocytes, regulatory T cells, immune checkpoints, cytokines and cytokine receptors, human leukocyte antigens, and proinflammatory and para-inflammations. Moreover, a number of p53-mediated pathways are significantly associated with immune activities. These observations are confirmed in another cohort in which a low expression of PD-L1 and reduced number of infiltrating lymphocytes correlates with the *TP53* mutation [103]. The level of PD-L1 seems to be particularly lower in tumors with a *p53* mutant and elevated *HER2* expression [104]. As an immunotherapy, anti-PD-1 immunotherapy shows a limited efficacy in GC; these observations may open the way for a therapeutic strategy based on a combined use of a p53 reactivator and trastuzumab (anti-HER2). In addition, *p53* mutants promote a hypoxic response in GC tumors, which likely impacts the immune response [88]. 

### 3.6. Is the *TP53* Expression Level a Biomarker for Personalized Therapy in Gastric Cancer?

Another approach often used in pathology for establishing a prognosis uses the protein expression level, independently of the mutation status. Drs Martin and Sylvester were the first to take advantage of Dr. Lane’s p53 monoclonal antibodies and investigate by immunohistochemistry the correlation between the p53 protein level and a GC patient’s prognosis [105]. This initial study found that 57% of the tumors expressed a high level of p53 protein and that these patients had a lower five-year prognosis (24% vs. 56% with low level of p53 proteins; *p* = 0.02, Mantel-Cox Test; odds ratio of death, 2.09; 95% confidence interval I 0.02 to 4.25; *n* = 126). A high expression of p53 was present in the intestinal (62%) and diffuse (48%) types of GC, including the signet cell type (71%), and lymph node metastases (64%). Similar results were later confirmed on larger cohorts (*n* > 200), showing that the elevated p53 expression could be an independent factor for a poor prognosis in patients that undergo curative resection [106] or in general, with a significant difference in the five-year survival rate (62% vs. 43%) [107], and that a positive nuclear p53 immunoreaction can be significantly associated with a shorter overall survival [108,109]. It was also shown that GC displaying the combination of a high p53 expression and aneuploidy carries the worst prognosis [110].

However, these results on the prognostic value of p53 protein expression were contradicted and revealed a more complex picture [111,112,113,114,115,116]. For instance, some studies identified p53 expression as a poor prognosis marker only in the diffuse subgroup [117] or only in a univariable analysis (*p* < 0.0005) but not in the multivariable analysis, although relatively close to be statistically significant (ex., *p* = 0.08) [81,115,118].

These discrepancies illustrate the complexity of GC and the difficulties encountered in studies differing by the patients’ profiles, the antibodies used, and the cut-off scores chosen for positivity, as well as the statistical methods. In particular, these studies did not analyze the mutational status of *TP53*; thus, the relative representation of the *TP53* mutations between the cohorts might explain some differences in the outcomes. Hence, several meta-analyses have been performed and concluded that a high expression of p53 proteins should be considered a biomarker for a poor prognosis (ex., [119]). Therefore, an elevated p53 protein level represents an indicator of a poor prognosis for GC patients.

In addition to the patient prognosis, multiple studies have shown that a high p53 expression was a marker for a weak response to chemotherapy [120,121,122,123,124]. For example, a study combining three large cohorts (1150 patients) indicated that p53 expression (DO7 antibody) was a predictive marker for survival (*p* < 0.001) and a response to therapy (5-FU+folinate+oxaliplatin, *p* < 0.01) [125], but such a correlation was not always observed [126]. However, a meta-analysis of more than 800 articles concluded that the p53 expression should be considered an interesting biomarker for the therapy response [86,127].

To increase the selectivity and sensitivity of the prognosis and therapeutic prediction, the co-expression of p53 with known positive or negative regulators of the cell cycle and apoptosis have been investigated in GC. For instance, the elevated co-expression of p53 with BCL2 [128], TS GSH [129], or VEGF [130] correlate with a poor response to 5-FU and cisplatin. Similar results were obtained for an association between p53 and MCL1 [131], Cyclin E [132], HIF1A [133], or MMP7 [127]. In addition, an inverse correlation was found between the p53 and Survivin expression [134], which is repressed directly by p53 [135].

Besides establishing a correlation between the p53 protein expression and survival, investigators have tried to define the characteristics of the tumors expressing high levels of p53. For instance, the nuclear accumulation of p53 protein was frequently seen in papillary adenocarcinoma, well-to-moderately differentiated tubular adenocarcinoma and poorly differentiated adenocarcinoma with solid nests or focal tubular structures but was rarely seen in signet-ring cell carcinoma, mucinous adenocarcinoma, or poorly differentiated adenocarcinoma growing in a scattered manner [136,137]. In a French cohort, similar results were observed, showing that 67% of the intestinal type of GC but only 24% of the diffuse type express high levels of nuclear p53 [138]. There were also differences in the p53 expression between proximal and distal CG, respectively, 38% vs. 20% [139]. A similar observation was made for *MDM2* and *p14-ARF* [140]. In addition, *H. pylori*-infected GC show higher p53 staining [141], whilst the eradication of *H. pylori* reduces the p53 and *MDM2* protein levels [142]. High levels of p53 expression occur in primary and metastatic GC, supporting a correlation with elevated aneuploidy and the proliferation rate [137,143,144]. In contrast, the absence or low expression of p53 was observed in the very early stage of gastric carcinogenesis, such as chronic gastritis or metaplasia [145,146]. The expression of p53 appears to increase with the carcinogenesis [147]. However, some studies did not see the same correlation between a high p53 expression and cancer stage [114]. 

One of the difficulties is to make a precise assessment of the p53 status, mutation and expression-wise. The studies described in this section did not assess the mutational status of p53 in parallel to the expression level. A plausible hypothesis is that the elevated p53 protein level could be explained by the presence of a mutation in the p53 that would inhibit its transcriptional activity, hence reducing the expression of the main p53 repressor and target gene the *MDM2* responsible for p53 degradation. Thus, the p53 expression and *TP53* mutations might be considered equally concerning the p53 status. However, several studies have shown discrepancies between the expression level of p53 and the *TP53* mutation status in GC [32,148,149,150]. For instance, a third of the tumors with high p53 expression have no *TP53* mutation and vice versa. Hence, studies considering only the expression level have to be taken cautiously. Overall, assessing both, the protein expression level, as well as the mutational status, seem to have a clinical interest, since accumulation of the wild-type p53 protein in tumors correlates with a lower risk of metastasis [151]. 

To simultaneously assess the expression level and the mutation status of *TP53*, an interesting approach is to test for the presence of autoantibodies directed against p53 in patients’ blood. Several studies, including meta-analyses, showed that the presence of p53 autoantibodies correlated with the stage and prognosis [152,153,154,155,156,157] but were not always confirmed [158]. More recently, the detection of p53 autoantibodies combined with other autoantibodies has been evaluated with success to diagnose GC [159].

Hence, to summarize, an elevated expression level and/or mutation in p53 should be considered as prognosis and predictive markers in GC, despite some contradictory results. These contradictory results may also reflect the complexity of GC and may also justify why, in the particular case of GC, the status of p53 (mutation or expression level) is not used in current clinical practice to stratify the patients and make a reasoned therapeutic decision. Another explanation is that there is a lack of alternative therapies to treat GC if chemotherapy is not used. 

### 3.7. Alteration of p53 Regulators in Gastric Cancer

When no *TP53* mutation is present, inhibition of the p53 function can also occur by alternative means that impact negatively on the p53 protein level and/or activity, favoring tumorigenesis. Multiple mechanisms have been described in GC—in particular, alterations that target the expression or activity of MDM2. 

#### 3.7.1. *TP53* and *MDM2* Alterations in Gastric Cancer

The E3 ubiquitin ligase *MDM2* is the major negative regulator of p53 through a direct interaction that drives p53 ubiquitination and degradation by the proteasome. *MDM2* does not seem to be overexpressed at precancerous stages [160], but *MDM2 amplification* was found in 42% of GC and in a majority of the diffuse type [47]. A detailed analysis of the TCGA data shows that 20% of GC display high *MDM2* mRNA levels or gene amplification in both the *intestinal* and *diffuse* types (personal data). Surprisingly, the gain or overexpression of *MDM2* is similarly found in tumors with WT or mutated p53 (24%, personal data). Interestingly, tumors infected by *EBV* display significantly more *MDM2* mRNA. In addition, a *polymorphism* (SNP309 G/G) in the *promoter of MDM2*, which induces *MDM2* transcription, correlates with an increased risk of GC by hazard ratio, 3.16; 95% CI, 1.22 to 8.20; *p* = 0.018 [161,162]. The elevated expression of *MDM2* in GC is also caused by the loss of *miR-518* that targets *MDM2* [163].

Besides the alterations of the *MDM2* gene, other molecular mechanisms that affect the *MDM2* protein activity have been described in GC. The elevated expression of positive *MDM2* regulators—namely *MDM4* (*MDMX*) [164], *ENIGMA* [165], *AURKA* [166], and *CDT2* [167]—correlates with the *MDM2* protein level and has an impact on the prognosis. For instance, *MDM4* has an elevated expression that correlates with lymph node metastasis [164]. Inversely, the downregulation of *MDM4* increases slightly the activity of cytotoxic drugs (5-FU) in GC cells [168]. Another example is the LIM domain protein *ENIGMA*, whose overexpression is caused by *SRF* [165]. At the molecular level, *ENIGMA* interacts with *MDM2* to inhibit its self-ubiquitination, thereby stabilizing it and inducing p53 ubiquitination and degradation. Similarly, the *Aurora A kinase (AURKA)* stabilizes *MDM2* by phosphorylation at *S166* to reduce the p53 protein levels by ubiquitination [166].

Conversely, several negative regulators of *MDM2* are lost in GC. For example, a mutation of *SMAD1* at *S239* found in GC favors the interaction of *MDM2* with p53 by avoiding the phosphorylation of SMAD1 by ATM, a DNA damage response kinase, thereby reducing the cell death caused by doxorubicin or *BMP2*, a member of the *TGF-β* family [169]. However, the frequency of this *SMAD1* mutation seems to be very low, as a *TCGA analysis* on a cohort with 478 patients did not show any mutation and, rather, showed a selective increase in its expression in the singlet cell GC subtype (personal data). Another example is the reduced expression of *PICT1* in GC that correlates with a better prognosis (*n* = 70 patients; *p* = 0.046) [170]. At the molecular level, *PICT1* appears to sequester the ribosomal protein RPL11 in the nucleus to destabilize MDM2. *UBTB1* also has a reduced expression in GC that correlates with a poor patient survival (*p* = 0.012, stage I/II). This ubiquitin ligase induces the degradation of MDM2 to stabilize p53 and induces senescence [171]. The expression of *FHIT* is mainly lost in diffuse GC that rarely display *TP53* mutations [172]. *FHIT* (fragile histidine triad), a P1-P3-bis(5′-adenosyl) triphosphate hydrolase, is a tumor suppressor gene frequently lost in various cancers that inhibits the MDM2-dependent degradation of p53 [173].

#### 3.7.2. Other p53 Regulators Altered in Gastric Cancer

Besides molecular alterations that affect *MDM2*, other silenced or overexpressed genes in GC alter the p53 activity and impact the prognosis. A few of these genes have their expression diminished by promoter methylations. For instance, the promoter methylation of *PAX5* [174], *OSR* [175], *PKNOX2* [176], and *BCL6B* [177] occurs in GC, and the restoration of their expression, by overexpression or treatment with *5-Aza*, increases the p53 activity on *P21*, *BAX*, *FAS*, or *IGFBP5* expression. Importantly, the methylation of *OSR* correlated with a poor prognosis in a multivariate analysis (*p* = 0.04). Another example is the loss of a positive regulator of p53, *TIP30*, that correlates with a reduced survival in GC patients [178]. Its re-expression in GC cells restores the p53 protein levels and induces the expression of the proapoptotic target gene of p53 *BAX*. 

Conversely, the elevated expression of p53-negative regulators is also observed in GC, such as *ubiquitin ligase* TRIM59, which is upregulated in the T2 and T3 stages and correlates with a poor prognosis (*n* = 111, *p* = 0.447) [179]. *TRIM59* interacts with and drives p53 ubiquitination, whilst *TRIM59* silencing reduces the cell growth and migration that correlates with upregulation of the p53 protein and *NOXA* mRNA levels. Other examples are the HSP70-type protein CREP that interacts and negatively regulates p53 [180] and *ZBTB2*, whose expression is increased in GC due to the loss of *miR-149* expression [181].

The deregulation of additional genes that correlate with p53 activity have been described, such as the low expression of *CARP* (caspase-associated recruitment domain-containing protein) [182], *Ku70*, and *CHK2*, which leads to impairment of the DNA damage response (DDR) mechanisms [183], *Ubiquilin 4 (Ubqln4)*, encoding a ubiquitin ligase interacting with RNF114 [184], and *CHD5* (chromodomain helicase DNA-binding) [185]. Inversely, the elevated expression of *AP-4* [186], the bHLH-LZ interactant of the large T antigen of SV40 *Mortalin* [187], *DITT4* [188], *Mucin 17 (MCU17)* [189], *PDRG1* (P53 and DNA Damage-Regulated Gene 1) [190], *MPP8* (M-phase phosphoprotein 8) [191], *HBXIP* (Hepatitis B X-interacting protein) [192], *IRTKS* [193], and the Cdc42 interacting kinase gene *ACK1* [194] correlates in GC with a reduced p53 activity, and their silencing restores p53 functions characterized by cell growth arrest or cell death. However, the precise mechanisms connecting the change in p53 activity to the deregulation of these genes and their impact on patient prognosis has not yet been established. 

Another mechanism that impacts p53 expression involves *noncoding RNAs*. The downregulation of *miR-370* [195] increases the expression of its target, *PTEN*, which, in turn, inhibits p53. Inversely, the elevated expression of *miR-27b* [196] or *miR-100* [197] downregulates the expression of p53 activators *CCNG1* and the ubiquitin ligase *RNF144B* (an inhibitor of the p53-ubiquitin ligase *PIRH2*), respectively. Several long noncoding RNA (*lncRNA*) also induce the expression of p53 in GC. For instance, *MEG3* *lncRNA* increases the p53 protein levels to mediate its antitumor activity [198]. Its elevated expression results in a good prognosis for GC (*n* = 72, *p* < 0.001), but its expression can be downregulated by methylation. Similarly, *GAS5 lncRNA* stabilizes p53 in GC cells by interacting with *TP53* exon 12, reducing the proliferation and migration [199]. Inversely, several oncogenic lncRNA (*OnclncRNA*) can inactivate the p53 pathway to promote gastric tumorigenesis, such as *OnclncRNA-626* through an interaction with *SRSF1* [200], *VCAN-AS1 lncRNA* through a competitive interaction with eiF4A3 that correlates with a poor prognosis [201], and *lncRNA ZFPM2-AS* via the stabilization of the *MIF* (macrophage inhibitory factor) [202]. Finally, the circular RNA *Hsa_circ_0001546* acts as a *miRNA-421* sponge to inhibit the chemoresistance of GC cells via an ATM/Chk2/p53-dependent pathway, correlating with a poor prognosis [203]. 

### 3.8. Alteration of p53 Target Genes in Gastric Cancer

Alterations found in GC also impact the downstream components of the p53 pathway. More precisely, the known p53 target genes involved in cell growth or death are altered. In several cases, the change in expression is most likely due to the presence of p53 mutations, such as for the elevated expression of *HDGF*, which is normally repressed by the binding of WT p53 to its promoter [204]. Inversely, the expression of *RHOE* [205] and *FBXW7* [206] is low in GC, while the overexpression of p53 restores their expression. Surprisingly, the p53 target gene *TIGAR*, involved in the cell metabolism, has an elevated expression in GC, where it protects cells from *RAS* and favors cell proliferation [207]. Hence, the expression of *TIGAR* in GC appears independent from the p53 status. Noncoding RNAs are also impacted. For instance, the *lncRNA TUSC7* is a direct target of p53, whose expression is inhibited in GC and correlates with a poor prognosis (*n* = 78, disease-free *p* = 0.0019) [208]. Similarly, p53 can directly induce the expression of *miR-508-5p* in GC cells, which targets *ABCB1* mRNA to favor the response to therapy [196]. However, there are also more interesting examples, such as the p53 target gene *BGT4* that is repressed through promoter methylation (66%), and the restoration of its expression using 5-Aza-DCT induces cell death and reduces tumor growth [209,210]. Another example is the mutations in the p53 target and pro-cell death receptor *DR5* that are exclusive of *TP53* mutations [211]. 

Overall, it is interesting to note that there are only two examples of alterations of the p53 target genes *BTG4* and *DR5* that are independent from the p53 status. This highlights how p53 seems to represent a key node involved in gastric epithelial cell homeostasis, which is supported by a study identifying a *signature* of p53 target genes that correlates the most with patients’ survival [212].

### 3.9. Functions and Regulations of p53 in Gastric Cancer

#### 3.9.1. Cellular and Molecular Activities of p53 in Gastric Cancer Cells

In 1991, Baker and Vogelstein showed that wild-type p53 could inhibit cell growth [8]. It was soon after confirmed that wild-type p53 inhibits growth of GC cells but not mutated p53 V143A [213]. The evidence of a dominant negative activity of the p53 mutant over *WT* p53 was also pointed out in GC, since half of the *TP53* mutations (54%) occurred in samples without allelic loss [214]. However, the role of p53 in response to DNA damage [215] and chemotherapy [216] was, at first, unclear, as an initial report suggested that *WT* *p53* was not induced by *these stresses* in MKN74 cells and did not participate in their response. However, it is of note that a lower band around 44 kDa detected by the p53 Pab1801 antibody was significantly induced and correlated inversely with the protein level of *BCL2*, an anti-apoptotic gene known to be repressed by p53. At that time, the nature of this band was unknown, but now, we can speculate that it could correspond to the p53 splice variant, *p53β*, which was described in GC cells treated with *cisplatin* [217]. The function of *p53β* in the GC cells remains to be established.

Since then, multiple studies have indicated that p53 is involved in the response to chemotherapy in GC cells. Typically, cell lines with mutated p53 (SK-GT1-5 and MKN28) are less sensitive towards 5-FU, cisplatin, or mitomycin C compared to cells that express WT p53 (MKN74, NUGC-4, and MKN45) [218,219]. Furthermore, the reintroduction of WT p53 in mutated p53 cells favors apoptosis induced by cisplatin or 5-FU [220] via the downregulation of AKT phosphorylation [221]. As observed in other biological systems, these drugs (ex., 5-FU) induced a stabilization of WT p53 and a change in expression of the p53 target genes, such as *BCL2* and *BAX* [222,223], but not mutated p53 [218]. 

These observations confirmed the initial findings made by Kastan et al. that p53 plays a role in the DNA damage response [224] and that the introduction of WT p53 in p53-null cancer cells favors a response to *cisplatin* in vivo by inducing apoptosis [225]. However, it is of note that most of the studies performed on GC cells based their conclusion on a correlation rather than on functional experiments. In 2000, Drs. Jiang and Lam were the first to use the *p53 loss of function (antisense)* in AGS cells to show that p53 was required to induce apoptosis caused by arsenic trioxide [226]. More recently, the function of p53 in DNA damage and chemotherapy responses in GC cells was confirmed by showing that the silencing of p53 and ATM synergize to favor the cytotoxicity induced by an inhibitor (Olaparib) of the DNA damage response enzyme *PARP-1* [227]. However, the demonstration that the *mutational status of p53* dictates the response to *chemotherapy* in *mouse models* of GC remains to be established [228]. 

The activity of p53 upon treatments has been assessed by the regulation of well-known *p53 target genes*, such as *P21* and *BAX*. However, other target genes have been also investigated. For instance, chemotherapies (cisplatin, 5-FU, etc.) can induce the promoter activity of the receptor for pro-death ligand FAS *CD95* via p53 in GC cells [229,230]. In contrast, p53 can repress the *TFF2* gene, involved in wound healing in the digestive tract, by competing with the AP1 transcription factor, inhibiting cell migration and inducing apoptosis [231]. In contrast, mutant p53 R175H favors *TFF2* expression. Similarly, P53 inhibits the E2F1-dependent induction of *CHK1* upon radiation treatment in AGS cells, explaining why high level of nuclear CHK1 correlated with a poor prognosis in GC [232].

Numerous other mechanisms positively (ex., CHK1/2 kinases) and negatively regulating p53 (ex., MDM2) have been identified in cancers. Only a handful of them have been characterized in GC. More precisely, *hSRBC*, a putative tumor-suppressor gene seems to be an inducer of p53 in response to chemotherapy (5-FU, etoposide) in GC [233]. The topoisomerase TOP3a is required for p53 activity through a complex mechanism involving its binding to the p53 promoter and a physical interaction with the p53 protein [234]. Hence, growth inhibition induced by TOP3a requires p53, and therefore, *TOP3a* expression is logically found downregulated in GC. Inversely, *XIAP* seems to be a p53 inhibitor reducing apoptosis caused by chemotherapy (ex., cisplatin) in p53 WT GC cells [235]. Other examples are a diminished expression of p53 by *miR375* by interacting with the p53 3′UTR [236] or the inhibition of *PI3Kcat* leading to cell death that correlates with an increased expression of *p53* and *PUMA* [237]. However, the significance of these mechanisms for the patient outcome has not been established yet.

Beside chemotherapies, other drugs and stresses inducing growth arrest or cell death have been described to modulate the p53 activity in GC cells. *TNFα* induces apoptosis via an increased p53 expression, which, in turn, induces *BAX* [238] and *IGFBP3* [239] expression. *TRAIL* (TNF-related apoptosis-inducing ligand) is more active in MKN45 cells expressing WT p53 [240]. Besides these proapoptotic ligands, the activation of *PPARg* by troglitazone induces apoptosis via p53 and the downregulation of *BCL2* in GC cells [241] [242]. Inversely, p53 is inhibited in GC by persisting *pollutants* (ex., *PFDA*) [243].

P53 has also been a marker to explore the potential of novel anticancer therapies. For instance, the p53 and *BAX* protein levels are induced by *S-propargyl-cysteine (SPRC)*, which causes *H_2_S* production by the *CSE* enzyme of the GSH metabolism pathway (trans-sulphuration pathway) and cell death [244]. *Copper ternary complexes* with amino acid chains [245] or *iron chelators* (ex., DpdtC (2,2′-di-pyridylketone dithiocarbamate) [246] induce p53 activity in GC cells. Finally, several molecules derived from *Chinese medicine*, such as *Pteris semipinnata L*, induce cell death more efficiently in GC cells with WT p53, [247,248], involving the inhibition of *MDM2* in the case of *triptolide* [249]. 

However, p53 does not seem to be involved in the response to all pro-cell death signals in GC cells. For instance, TGF-β causes apoptosis in GC cells by a p53-independent mechanism [250,251]. However, TGF-β alterations (20% mutations in the TGF-β RII receptor) are exclusive to *TP53* mutations in GC and correlate with the microsatellite instability [33]. In addition, the genetic inactivation of the TGF-β signaling component, *SMAD4*, does not favor *TP53* mutations in mice while inducing gastric polyps [252]. Hence, this would suggest that p53 and TGF-β either participate in the same molecular pathway in gastric tumorigenesis or that p53 inactivation is not necessary for the tumorigenesis of the MSI subtype. Other drugs or stresses were shown to act independently of p53, such as *cadmium* [253], *paclitaxel* [254], *cetuximab* [255], *arsenide tetroxide* [256], and *ruthenium complexes*, representing interesting molecules to treat p53-mutated GC [257,258]. This is the case for nonsteroid anti-inflammatory molecules (*NSAID*) (ex., indomethacin) that target the COX2 enzyme and display anticancer activities. Several p53-target genes, such as *PUMA*, are activated by NSAID [259], but NSAID anticancer activities seem to be independent of p53 in GC [260,261] and might involve *ER stress pathway* effectors (*ATF4* and *CHOP*) instead [262]. Still, there is a functional interaction between p53 and COX2, since the expression of *COX2* is repressed by p53, which competes with *TATAA-binding proteins* on the *COX2* promoter [263]. This may explain why the expression of *COX2* is more elevated in GC with *TP53* mutations [264]. Hence, targeting *COX2* might represent an interesting strategy in tumors with *TP53* mutations that are often more resistant to chemotherapies.

#### 3.9.2. p53 Mouse Genetics and Gastric Cancer

P53^−/−^ null mice are viable but were shown to be highly susceptible to spontaneous tumorigenesis, with a predominance of malignant lymphomas, osteosarcomas, and soft tissue sarcomas [265,266]. To our knowledge, no spontaneous tumor formation in the stomach has been reported in p53^−/−^. However, when p53^−/−^ null mice are treated with the carcinogen N-nitrosomethylbenzylamine (*NMBA*), about 50% of p53^−/−^ null mice develop tumors in the stomach. Interestingly, zinc depletion makes these mice more susceptible to *NMBA*, and 100% of p53^−/−^ null mice develop tumors within 30 days [267]. This suggests a role for p53 in regulating *zinc* metabolism in the stomach, as seen in HEPG2 cells [268]. Hence, the loss of *tp53* may be a facilitator for the early step of gastric tumorigenesis, which is supported by the observation that gastric organoids are deleted for *tp53* to evolve toward dysplasia and then adenocarcinomas when implanted in vivo [269]. In the following years, many other groups combined p53^−/−^ null mice with other mouse models to induce and study tumorigenesis in the stomach. Importantly, these studies revealed that, depending on which mouse model(s) are combined with p53^−/−^ null mice, tumorigenesis can be directed towards the development of intestinal carcinoma, diffuse with the presence of ring cell carcinomas, or a mixed type of GC.

More precisely, when p53^−/−^ null mice are combined with

(1) the loss of *cdh1*, mice develop poorly differentiated GC with the presence of ring cell carcinomas [270].
(2) In addition to the loss of *tp53* and *cdh1*, the loss of *smad4* drives the development of the diffuse type of GC with the presence of signet ring cell carcinomas [271]. In this model, the osteopontin cytokine, Zeb2, and thymosin-β4 (Tβ4) were later identified as relays for tumor formation [272].
(3) Replacing, in the latter case, the *smad4* loss by the gain of expression of the oncogene *kras^G12D^*, generating a compound mouse with the loss of *tp53* and *cdh1* and gain of the *kras^G12D^* oncogene expression results in the development of diffuse and intestinal type of tumors [273].

These different models clearly show that, by combining the loss of *tp53* with the loss of *cdh1* and *smad4* or gain of *kras^G12D^*, one can drive gastric tumorigenesis either towards a diffuse type of gastric cancer or mixed type or diffuse and intestinal. However, the major drawback of these models is that they are not time-controllable, as the Cre driver lines used are already active at the embryonic stages. In addition, they seem to be not active in all cell types of the gastric epithelium, and/or as in the case of *pdx1-Cre*, which are not restricted to the gastric epithelium. Instead, they induce tumors in other organs, like the small intestines or the pancreas. Ideally, one would need a mouse model where GC can be induced at adult stages specifically only in the stomach and where the tumor development is not only directed towards diffuse or mixed types of tumors but, more precisely, towards the CIN, GS, EBV, and MSI types of gastric adenocarcinomas. 

In this respect, Seidlitz and coworkers set out to fulfill these needs [274] by taking advantage of various mouse tissue gene expression databases (Gene Expression Omnibus, MGI-Mouse Gene Expression Database (GXD), and BioGPS) to identify a stomach-specific gene to drive a tamoxifen-inducible Cre recombinase (CreERT2) and improve our knowledge concerning the genetic characteristics of the different GC subtypes. Concerning the first aspect, they identified the *anxa10* gene locus to be specifically expressed in all cell types of the gastric epithelium only and generated the tamoxifen-inducible anxa10-CreERT2 driver mouse line. Combining anxa10-CreERT2 mice with

(1) Tp53^R172H^ + Kras^G12D^ + Smad4^fl/fl^ drove gastric tumorigenesis towards the CIN type with intestinal morphology, glandular structures, and the development of lung and liver metastasis.
(2) Cdh1^fl/fl^ + Kras^G12D^ + Smad4^fl/fl^ gave rise to a diffuse GS type of gastric adenocarcinomas with diffuse morphology, poorly differentiated, signet ring cells, and peritoneal carcinomatosis and lung metastases.
(3) Cdh1^fl/fl^ + Kras^G12D^ + Apc^fl/fl^ gave rise to the newly identified GS subtype “Serrated adenomatous type cancer” with serrated tooth like morphology and adenomatous morphology [275].

Importantly, by using CreERT2, they could induce GC at an adult stage, permitting them to follow the tumorigenesis over time. Taken together, their mouse models hold many promises, but one has to wait to see if they will also succeed in generating EBV and MSI GC subtype mouse models.

In addition to these sophisticated mouse models, other published models have also shown the role of p53 in the tumorigenesis of GC. For example, heterozygotes mice for *tp53*^+/−^ and *lkb1*^+/−^ develop more polyps in the pylorus region than *lkb1*^+/−^ mice only, 92% vs. 69%, respectively, indicating their cooperation in GC development [276]. The deletion of *tp53* and *rb* in LGR5/CD44 cells strongly induced the development of gastroesophageal carcinomas in the gastric epithelial junction and a low frequency (10%) of benign tumors, adenomas, in the antral region [277]. Transgenic mice expressing mutant *p53* *V143* develop a higher percentage of GC (20% vs. 4%) when treated with 4-nitroquinoline-1-oxide [278]. As expected, this increased tumorigenesis correlates with a decreased expression of genes involved in cell cycle inhibition and apoptosis. Interestingly, it also correlates with a decrease in genes involved in inflammation (ex., *tnfa* and *cxcl9*) and the immune response (ex., *irf1*, *3*, and *7*). Finally, the deletion of *tp53* in a *wnt1*-activated background induces the EMT (Epithelial Mesenchymal Transition) and gastric epithelial cell transformations with the cooperation of the *microenvironment* via COX2 activation [279]. Although not directly addressing the role of p53 in GC, Guo and coworkers demonstrated that the loss of *pten* (SP-A-Cre; pten^flox/flox^) induces differentiated GC within 80 days depending, in part, on the expression of *p53* [280]. More precisely, they demonstrated that the loss of *pten* leads to an upregulation of Akt activity, which represses the p53 expression, resulting in a decreased expression of miR-365, as it is a target gene of p53. Consequently, there is an increased expression of two of its target mRNAs: *cyclin D1* and *cdc25* and enhanced proliferation. Relevantly, the miR-365 expression is reduced in GC in patients, which inversely correlates with the *pten* expression (r = 0.68; *p* < 0.001).

Mouse genetics has brought interesting observations about the relationship between gastric tumorigenesis and mutations of *TP53*, pointing to their importance for the development of intestinal/CIN carcinogenesis. However, no information exists about p53 isoforms or mouse inactivated for p63 or p73 isoforms regarding gastric tumorigenesis, despite the fact that several studies pointed to their alterations in GC.

#### 3.9.3. Deregulation of the p53 Pathway by *H. pylori*

An infection with *H. pylori* is a major factor causing GC with intestinal and chromosomal instability characteristics (CIN) that predominantly display *TP53* mutations. In mice, p53 is a protector against *H. pylori*-induced proliferation during gastritis but does not seem to provide protection against GC development, possibly due to a reduced *Th1 immune response* [281,282]. In Mongolian gerbils, an infection with *H. pylori* induced a dynamic change of the p53 protein levels in the gastric tissues, peaking four hours after infection but decreasing to normal level after 12 weeks [283]. In vitro, an infection with *H. pylori* or expression of the *H. pylori* protein *VacA toxin* in GC cells inhibits cell growth, induces apoptosis, and causes a transient increased p53 expression at two hours with a subsequently progressive decrease up to 72h conjointly with the p53 target genes (*P21*) [284,285]. Inversely, the *p73* expression increases to reach a peak at 72h, but the identity of the isoform, TA or ∆N, remains to be characterized. 

The delayed reduction of the p53 protein level is caused by degradation involving multiple mechanisms. For instance, the *CagA* protein secreted by *H. pylori* induces mechanisms that destabilize p53—namely, the phosphorylation of *MDM2* by AKT [283], the induction of an ARF-BP1 ubiquitin ligase complex [286], and the subversion of ASPP2 to target p53 for degradation [287]. Inversely, *H. pylori* represses the expression of the mechanisms that stabilized p53, such as *E-cadherin* that destabilizes the p53/MDM2 interactions [288], *USP7* that deubiquitinates and stabilizes p53 [289], and *USF1* (promoter methylation) that reduces the USF1 ability to interact with and stabilize p53 in the nucleus by competing with MDM2 [290]. Typically, the silencing of *E-cadherin* by siRNA restores the p53 expression that correlates with the induction of *NOXA* and *P21*. The importance of the p53/MDM2 *interaction* in *CagA* activity is illustrated by the fact that the inhibitor of the p53/MDM2 interaction, *Nutlin-3,* restores the p53 protein level after CagA exposure. 

Besides affecting the p53 expression, a *H. pylori* infection increases the expression of the p53 isoform *∆133p53* at the transcriptional level via an *AP1*-binding site targeted by *c-Jun* [291]. The expression of *∆133p53* inhibits the activity of p53 and p73 and induces the NF-kB pathway (p53 expression and target genes *IL-6, IL-8*, and *BCL2*) by inhibiting the *IKK* expression to reduce apoptosis. 

#### 3.9.4. Deregulation of the p53 pathway by the Epstein–Barr Virus

The Epstein–Barr virus (EBV) is associated with several human malignancies, including GC [292,293,294]. Infection by the EBV defines a specific molecular subclass that represents about 9% of GC (Figure 1). However, it has been suggested that the EBV subgroup might be heterogeneous. For instance, EBV infection (detected by EBER staining) is of poor prognosis (Hazard ration, HR 2.9) in GC with the intestinal characteristics and of good prognosis (HR 0.4) in GC with diffuse characteristics [295]. However, EBV tumors have no *TP53* mutation, and the EBV infection (viral *EBER1* mRNA) is homogeneously present in the tumor, suggesting that the infection is an early event in the carcinogenesis process [296,297,298,299]. If EBV infection is exclusive of *TP53* mutations, in contrast to *H. pylori*, few of the 70 open reading frames (ORFs) contained by the 184-kb linear double-stranded DNA EBV genome, which code for latent and lytic viral proteins, can have an impact on p53 activity [293]. For instance, the EBV viral protein *EBNA1* downregulates p53 activity via USP7 (a p53 regulator) and *PML bodies*, reducing the response to DNA damage in GC cells [300,301]. In other types of EBV-induced cancers, additional viral proteins were shown to be involved in p53 inhibition. For instance, the EBV immediate early transcription factor BZLF1, which is the main regulator of the viral life cycle by controlling the switch between its latent and lytic stages, interacts with p53. This interaction allows the binding of p53 to the elongin BC–cullin 5–SOCS box ubiquitin ligase complex, causing p53 degradation independently of *MDM2* [302]. In addition, the latent phase viral protein EBNA3C interacts directly with the p53 at its C-terminus, reducing the binding of p53 to its target genes and some of its regulators, such as *MDM2* [303]. In addition to proteins, EBV encodes about 44 *miRNAs* and *circular RNAs*. Some target the p53 mRNA, such as *miR-BART5-3p* [304] and *miR-BART3-3p* [305]. The EBV-encoded circular RNA *ebv-circLMP2A* plays a crucial role in maintaining stemness phenotypes through targeting the *miR-3908*/TRIM59/p53 *axis*, significantly increasing the metastasis and correlating with a poor prognosis [306]. 

However, the relationship between *EBV* and p53 is more complex. In EBV latently infected cells, the LMP1 viral protein can promote p53 accumulation by reducing its ubiquitination mediated by MDM2 at two sites, K48 and K63, and the interaction with tumor necrosis factor receptor-associated factor 2 (TRAF2) [307]. These mechanisms may explain an increase in the p53 protein level despite a reduction of *TP53* mRNA in some EBV-infected GC [308]. In addition, the functional interaction between the EBV and p53 is reciprocal, as *p53/ATM* [309] and the hypoxia inducible factor *HIF1A* [310] participate in the *reactivation of the viral lytic phase* through their binding to a hypoxia response element (HRE) located within the promoter of the EBV latent-lytic switch *BZLF1* gene Zp.

Finally, it is of note that *EBV* is not the only virus associated with GC. Indeed, the lesser-known *JC virus* is also suspected to cause GC. In this case, the transformation process seems to involve the multifunctional viral protein T-Ag that inhibits p53 [311,312].

### 3.10. Interactions of p53 with Oncogenic and Antitumoral Signaling Pathways in GC

#### 3.10.1. Interplay between p53 and HIF1A/Angiogenesis Mechanisms

As p53 is a key player in the cellular stress response, its role in the response to hypoxia has been extensively investigated—in particular, its interaction with the hypoxia inducible factor HIF1A. HIF1A is a key transcription factor that allows the cancer cells to adapt to the hypoxia environment present within the tumor in order to stimulate neo-angiogenesis, via the induction of *VEGF*, and to redirect the oxidative metabolism towards a glycolytic metabolism—via the induction of *GLUT1*, for example. The relationship existing between *HIF1A* and p53 is complex and has been detailed in previous reviews (ex., [313]). In this review, we are focusing specifically on what has been observed in GC. In GC, several studies showed that a high p53 expression, possibly caused by *TP53* mutations, correlated with elevated *angiogenesis* (anti-CD34) and necrosis [314,315,316] and is indicative of a *poor prognosis* [133]. At the molecular level, *hypoxia* stabilizes HIF1A while reducing the p53 and VHL protein levels in AGS cells expressing WT p53 but not in MKN28 cells expressing mutated p53 [317]. This favors a resistance to 5-FU chemotherapy [318]. In addition, HIF1A favors NF-kB binding on the promoter of the antiapoptotic genes *cIPA1* and *A20*. However, p53 expression also affects *HIF1A*, as *p53 mutants* in GC cells cause a hypoxia response and favor cell growth [88]. 

In addition to hypoxia, other metabolic stresses impact p53. For instance, *deoxycholic acid (DCA),* an inhibitor of the PK2 enzyme, induces p53 protein levels in SGC-7901 cells, which correlate with a change in the *BAX*/*BCL2* ratio and cell death [319,320].

#### 3.10.2. Interplay between p53 and NF-kB

There is a complex physical and functional interaction between p53 and the NF-kB pathway, a pathway important to control the immune response, notably via interleukins and interferons. However, little is known of its interaction with p53 in GC. Initially, it was shown that an inhibitor of *NF-kB* nuclear translocation, *SN50*, can induce p53 and PUMA protein levels in GC cells [321]. More precisely, SN50 induces p53 at the mRNA level, which contributes to the induction of autophagy via *BECLIN 1* [322]. It was also shown that inhibition of the NF-kB inhibitor, *IKK2*, leads to cell death and reduced migration in GC cells in vitro and in vivo, which correlates with induction of p53 and caspase 3 cleavage [323]. Inversely, STAT3, an activator of the NF-kB pathway, is a resistance factor toward chemotherapy in GC cells [324]. These studies indicate that NF-kB can repress p53 expression and activity in GC, at least partly, but NF-kB can also induce p53. In the MKN45 cells, *5-FU* can induce NF-kB, and the silencing of a NF-kB family member *RELA* reduces the p53 protein levels and expression of p53 target genes (ex., *PUMA*) [325]. Supporting the possibility that NF-kB may upregulate p53 is the finding that an elevated expression of *miR18a* in GC cells represses *IRF2* (interferon response factor 2), an activator of NF-kB [326], decreasing the p53 protein levels, leading to higher cell motility and correlating with a poor prognosis [327]. 

These data highlight the complexity of the interactions between p53 and the NF-kB pathway, likely reflecting the importance of the physiological context. Understanding these interactions is particularly crucial, as they may provide key information to explain how the *immune landscape* of GC expressing p53 mutants is deprived of immune cells compared to tumors with WT p53.

#### 3.10.3. Interplay between p53, the Hippo Pathway, and the Extracellular Matrix

The Hippo pathway has been widely described in different types of cancer to be altered and involved in tumor aggressiveness [328]. It is a mechano-transduction pathway that relays mechanical stress signals from the membrane towards the nucleus to drive adaptative gene expression. Hence, it has been connected to tight junction and extracellular matrix signaling. Several studies on GC have shown that the silencing of activators (e.g., *MST1/2*) of the Hippo pathway leads to nuclear accumulation of the oncogenic transcriptional cofactor YAP, favoring tumor aggressiveness and metastasis [329]. However, there are only two studies describing a functional interaction between p53, or a p53 family member, and the Hippo pathway in GC. The most interesting, however surprising, shows that mutations in p53, when associated with an elevated expression of the oncogenic YAP protein, favor GC patient survival [91]. The molecular mechanism mediating this counterintuitive association remains unclear. One explanation proposed by the authors is that the pro-proliferative activity of YAP and mutant p53 facilitates the activity of *cytotoxic drugs*. The second study suggested that a chemical, DIM, extracted from a Chinese vegetable has anticancer activity that correlates with an increased p53 protein level and activation of the *Hippo* pathway in GC cells [330]. Unfortunately, this limited information does not allow a good understanding of the functional interactions between the p53 family and the Hippo pathway in GC, highlighting the need for additional studies. 

However, several components of the extracellular matrix or the tight junctions, which interact with the Hippo pathway, can impact p53. For instance, *Cadherin 17* was found to be overexpressed in GC, and its silencing induces p53 protein levels to inhibit cell growth [331]. Similarly, the inhibition of *E-cadherin* expression by *H. pylori* induces p53 protein levels and the expression of its target genes (*NOXA* and *P21*) [288]. This inhibition is mediated by the *MDM2-ERK* pathway and the *H. pylori* CagA protein. In contrast, the *extracellular matrix* protein Periostin increases the p53 protein levels in GC cells, which is correlated with a decreased proliferation [332]. 

In summary, the activity of p53 seems to be regulated by extracellular signals caused by extracellular matrix components, tight junctions, and cell adhesion elements. However, whether this is mediated by the Hippo pathway remains an open question.

#### 3.10.4. Interplay between p53 and Epigenetic Effectors (HDAC, HAT, and MET)

The p53 protein has been shown to be the subject of multiple and complex post-translational modifications by several *epigenetic effectors*, such as *histone acetylases (HAT)* or *methylases*, which participate in the tight control of its activity [333]. Numerous studies have demonstrated that several of these epigenetic effectors are deregulated in cancers and can be targeted by various chemicals as potential therapeutic approaches. Surprisingly, only a small part of these findings has been investigated and confirmed in GC. For instance, the expression of p53 target genes (i.e., *P21*, *BAX*, *BAK*, *PIG3*, *NOXA,* and *PUMA*) is induced by two *histone deacetylase (HDAC) inhibitors (HDACi)*: *TSA* and *sodium butyrate,* in different GC cells [334,335,336]. This activation is mediated by acetylation at K320 and K373 on p53 [335], or the K382 following inhibition of *HDAC1,* induced by the silencing of *RNT* overexpressed in GC [337]. The induction of *PUMA* [336] and *DTWD1* [338] by *TSA* is *p53-* and *HDAC3-dependent*. A low expression of *PUMA* and *DTWD1* correlates with a poor prognosis, while *TSA* may represent an interesting therapeutic approach. However, *TSA*, as well as *sodium butyrate*, are pan-HDACi and, therefore, are not selective towards a specific *HDAC*, increasing the risk of side effects and/or complex antagonistic effects between different HDACs. Hence, more selective inhibitors have been developed. For instance, *HDAC4* has an elevated expression in GC, and its alteration (mutation or deletion) correlates with a better prognosis [339]. The inhibition of *HDAC4* by small chemicals enhances cisplatin-induced apoptosis in vitro and in vivo. This is partly mediated by p53, p73, and their target gene *BIK* [339]. Similarly, the elevated expression of the HDAC *SIRT1* in GC correlates with a poor prognosis (*p* = 0.008) and with a low expression of p53 [340]. The inhibition of *SIRT1* with Tenovin-6 induces p53 acetylation and *DR5* in GC cells [341]. 

The exact impact of *HDACi* on p53 protein levels is controversial, as an early study showed that, although it induces p53 target gene expression, *TSA* decreases the p53 protein level in GC cells [334]. Similarly, the silencing of *HDAC4* reduces the p53 protein levels [339]. However, another study contradicted these results by showing the synergistic effects of cotreatments between the *HDACi TSA* and *chemotherapies* (5-FU, oxaliplatin, and paclitaxel) to increase p53 expression in the MKN74 cells [342]. Another *HDACi, FK228*, also acts in synergy with the overexpression of p53, requiring *BAX* and *NOXA* [343]. 

The chemical inhibition or silencing of *methylases* leads to growth arrest or apoptosis in GC cells, and p53 seems to be involved, but the exact molecular mechanisms are still debated. For instance, the *pan-methylases inhibitor 5-AZA-cdr* induces cytotoxicity and p53 mRNA and the protein levels in MKN45 cells [344,345]. However, the reverse was also observed [346], as well as the involvement of a post-translational modification of p53 at S15 caused by *ATM* in AGS cells [347]. The selective inhibition (using siRNA or a selective inhibitor 3-Deazaneplanocin A, DZNep) of the *histone methyltransferase EZH2* of the *polycomb PRC2 complex* also induces cytotoxicity in GC cells via p53 [348,349]. At the molecular level, *EZH2* inhibition increases the p53 and MDM2 protein levels via the reduction of ubiquitination, inducing p53 target gene expression (*DR5*, *P21*, *FAS*, and *GADD45α*). In addition, the expression of EZH2 in GC inversely correlates with the activity of the p53 pathway (gene signature) [348,349]. Interestingly, the *histone demethylase JMJD2B* [350] is overexpressed in GC, and its silencing induces the p53 and P21 proteins in AGS cells, causing cell death in a p53-dependent manner. However, how the p53 protein level is regulated by this *histone methylase* is not known. Inversely, another *histone demethylase*, PHF2, interacts directly with p53 to induce its activity on its target genes (*P21* and *MDM2*) [351]. This also seems to have clinical relevance, since the expression of *PHF2* is downregulated or mutated in GC [352].

In summary, it appears that different inhibitors of HDACs, histone methylases, or histone demethylases can represent interesting strategies to treat gastric cancers. However, the molecular mechanisms involved are still largely elusive—in particular, regarding p53—handicapping their use for personalized therapy.

## 4. Developing the Magic Bullet to Target p53 Family Alteration in Gastric Cancer

**Figure 5:**
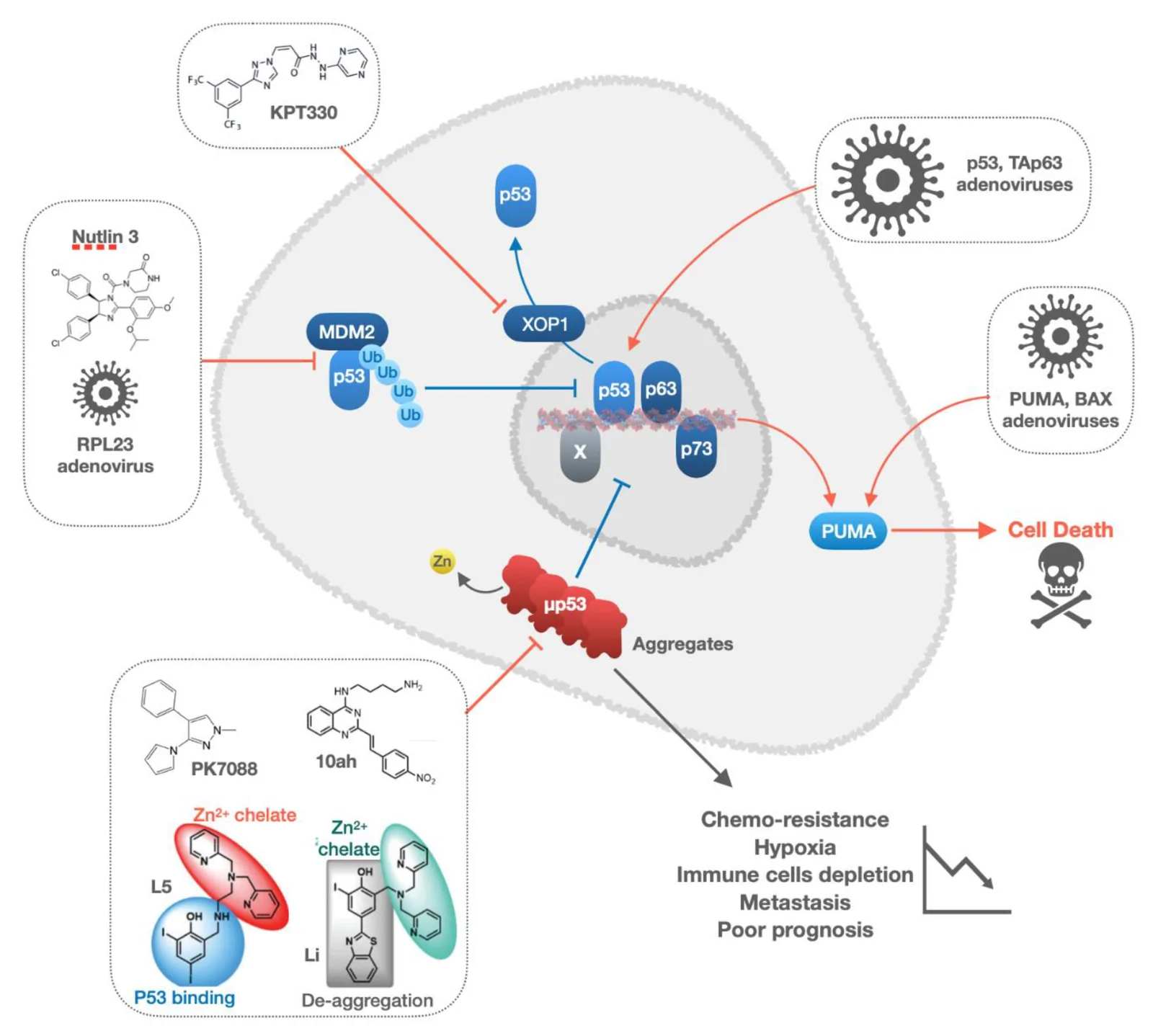
Therapeutic strategies targeting the p53 family that have been investigated in gastric cancer.

Based on its critical role in GC, p53 has been investigated as a potential therapeutic target. The first approach used in vitro and in vivo was the adenovirus transduction of WT p53 (*AdCAp53*) into MKN1 cells [353] (Figure 5). AdCAp53 induces growth inhibition and apoptosis in MKN1 p53-mutated GC cells but not in cells expressing WT p53. The activity of the transduced p53 can be increased by treating the cells with an HDAC inhibitor (Sodium butyrate) [354]. Importantly, AdCAp53 acts in synergy with chemotherapies (e.g., oxaliplatin) in vitro and in vivo [355,356]. Preclinical proof of concept has been published in GC, but this strategy (Gendicine^TM^ and Oncorine^TM^) is only used in China to treat head and neck cancers. Clinical trials for other indications are ongoing. As alternative, the transduction of an adenovirus expressing *the ribosomal protein RPL23,* which inhibits the interaction between p53 and MDM2, was performed in GC cells to induce cell death in vitro and reduce tumor growth in vivo [357]. Later, a bicistronic virus co-expressing *TP53* and *RPL23* was developed and showed a higher efficacy in vitro and in vivo than Ad-p53 alone, including in a GC orthotopic model [358]. An alternative to directly targeting p53 is to use downstream targets of p53. For instance, an adenovirus expressing *BAX* shows a high efficiency in killing GC cells, even if they are resistant to p53 overexpression [359]. Similarly, an adenovirus expressing *PUMA* under the control of the *β-catenin TCF-controlled promoter* significantly increases chemotherapy cytotoxicity in AGS cells in vitro [360]. The p53 family members have also been targeted. An adenovirus expressing *TAp63γ* (p51A) was shown to reduce the viability in GC cells (MKN45 expressing p63γ and MKN28 non-expressing p63γ) in vitro [361]. These p53 gene therapy approaches have been validated for some cancers in China, but they are not used in Western countries. 

Alternative strategies have been chosen in Europe and the US by focusing on small chemicals targeting p53 regulators or p53 mutants. For instance, the first inhibitors developed for clinical use were inhibitors of the interaction between p53 and *MDM2*, *Nutlins*, that bind to the p53-binding pocket of *MDM2*, resulting in p53 accumulation. The most common, *Nutlin-3,* induces a strong toxicity (IC_50_ ± 2–3 µM) in GC cells in vitro and in vivo [362]. In contrast, Nutlin-3 showed no cytotoxicity in mutated p53 cells (NUGC-3) up to 20 µM. Nutlin-3 stabilizes p53 and *MDM2* after four to eight hours of treatment (10 µM) in p53 WT GC cells but not NUGC-3 (p53 Y220C), leading to apoptosis. Nutlin-3 seems to have synergistic effect with cisplatin or 5-FU. In vivo, Nutlin-3 reduced MKN45 xenografts growth (−40%; 40 mg/Kg) slightly less than 5-FU (−60%; 40 mg/Kg), but combining Nutlin-3 and 5-FU blocks tumor growth (−80%). Nutlin-3 has been the leading compound from the Nutlin family and the base for the development of the derived compounds, such as *RG7112*, which has been tested in phase I for leukemias. Another MDM2-p53 interaction inhibitor, *APG-115*, induces cell cycle arrest and apoptosis in GC cells and acts synergistically with radiotherapy [363]. An alternative strategy is to inhibit *p53 nuclear export* by using *KPT330*, which targets *XOP1* (exportin1) often overexpressed in tumors and associated with a poor prognosis for patients [364]. KPT330 favors apoptosis of GC cells and acts synergistically with irinotecan to inhibit tumor growth in vivo.

The forced activation of p53 by inhibiting the p53/MDM2 interaction is an interesting approach, but it raises questions on the possible side effects on healthy tissues, since p53 is ubiquitously expressed. Hence, a promising approach consists of targeting p53 mutants selectively, as this may solely impact cancer cells, preserving healthy cells. However, the limit of this approach is that each mutation has a different impact on p53 structure and function [95]. Thus, a library of molecules able to target the various mutations is needed. For instance, p53 Y220C harbors a mutation in the cysteine that participates in an SH-stabilization of the Zinc, which forms the Zinc finger of the p53 DNA-binding domain. *PK7088*, developed by Drs. J. Spencer and A. Fersht, partially restored p53 Y220C activity in NUGC-3 GC cells when applied at high concentration (200 µM) [365]. To reduce the quantity of drugs required, we developed a bifunctional molecule able to interact with the mutant p53-Y220C and to restore zinc binding to p53 [366]. This molecule, *L5*, induces death in GC cells partly via reactivation of p53 Y220C function. However, it suffers from off-target effects, as cytotoxicity is also observed in cells expressing WT p53. This led us to optimize the structure and design novel compounds based on *L5* by targeting zinc loss and protein aggregation in p53 mutants. One of these compounds was found to be cytotoxic in GC cells expressing p53 Y220C while having no toxicity on healthy organoids [367]. Another type of molecules targeting p53 mutants, variants of *CP-31398*, have been tested in GC [368]. For instance, the variant *10ah* showed an interesting cytotoxicity in vitro and in vivo, reactivating the expression of *BIM*, *BAK,* and *BAX*. However, the selectivity of these complexes for the p53 mutant is relative, since additional mechanisms, such as reactive oxygen species, seem to be involved independently of p53 [369]. Another small molecule, *NA20*, a *naphthalimide* derivative, exhibits a selective growth inhibition of p53 mutant GC cell lines [370]. After coimmunoprecipitation and mass spectrometry (Co-IP/MS), it was found that *NA20* binds directly to p53 and is able to target DNA to trigger cytotoxicity. More precisely, *NA20* induces cell cycle arrest and apoptosis, which correlates with the activation of p21 and caspase 3/9 and inhibits tumor growth.

Altogether, besides the use of adenoviruses to induce p53, relatively little preclinical proofs of concepts have been provided for targeting the p53 pathway in GC models. In addition, some of the molecules tested have significant off-target effects. This highlights the necessity of further investigations using in vivo models and detailed verification of the mode of action of these strategies and the development of more selective compounds.

## 5. TAp73 and ∆Np73, the Yin and the Yang of Gastric Carcinogenesis

As a member of the p53 family, p73 presents two major types of isoforms (Figure 2). The *TAp73* isoforms have a transactivation domain in the N-terminal and are generally considered to be proapoptotic. The *∆Np73* isoforms lacking the N-terminus transactivation domain are considered to be anti-apoptotic with a dominant negative function over TAp73 and p53. These two types of isoforms are generated by alternative promoters [23]. Each of these two types of isoforms have additional variants in the C-terminus that are generated by alternative splicing. It is generally accepted that ∆Np73 has *oncogenic* properties and TAp73 has *tumor suppressor* function, such as induction of apoptosis in response to DNA damage [23,371]. However, these properties remain debated. Interestingly, the pro-apoptotic function of TAp73 can be inhibited by a direct interaction with p53 mutants [372]. Based on its role in the response to therapy and tumorigenesis, the alterations of p73 in GC have been investigated.

As observed in other types of cancers, p73 is very rarely mutated in GC [373,374]. For instance, our analysis of the TCGA indicates that 5 tumors out of 440 show mutations in *TP73*, located at A79T and A79V in the transactivation domain, P483L in the DNA binding domain, D611G just before the SAM domain and a non-sense mutation at position 205 within the DNA binding domain (personal data). These mutations are present in all molecular subgroups of GC, suggesting that their occurrence is not a consequence of the high mutational context of the MSI subgroup. However, the impact of these mutations on p73 function is unknown, but their presence in the DNA binding domain may drastically diminish p73 function, as seen for p53 or p63 [23]. Similarly, mutations in the transactivation domain might reduce p73 transcriptional activity. Beside somatic mutations, a polymorphism C4C14-to-A4T14 at exon 2 of *TP73* has been described in several cancers. This *polymorphism* does not seem to show any significant correlation with the risk of GC [375,376,377,378,379]. However, one study points out the possibility that the homozygous genotype favors GC of the intestinal subtype among the Caucasian population [380].

If p73 is rarely mutated, changes in its expression pattern have been observed in GC. A first detailed investigation indicated that p73 expression is detected in 50% of GC [381]. In addition, p73 has an elevated expression in *adjacent mucosa* of the tumors, suggesting a role in inflammatory tissues [382]. An elevated expression of p73 correlates with the loss of *Reprimo* due to methylation and lymph node metastasis [383]. Unfortunately, these studies did not clarify whether it was *TA* or *∆Np73* isoforms that were involved. A more detailed analysis showed that *∆Np73* can be found overexpressed in gastric tumors (62%, 15 out of 24) [384], which was confirmed by two additional studies [385,386]. This overexpression of *∆Np73* decreases the transcriptional activity of *TAp73* on reporter genes [387] and “typical” p73 target genes, but induces the expression of the *drug resistance* gene *MDR1*, via a direct binding of ∆Np73 on the *MDR1* promoter [385]. In contrast, *∆Np73* increases the β-catenin protein level and the activity of *TCF*-driven transcription. Importantly, *∆Np73* overexpression correlates with a *poor prognosis* in GC patients (20 months vs. 47 months) [386]. Hence, *∆Np73* seems to present *oncogenic* properties in GC and might impact on drug activity. 

At the molecular level, one mechanism explaining the elevated expression of *∆Np73* in GC is the loss of *HIC1* that negatively regulates the *∆Np73* promoter [386]. However, the correlation between *HIC1* and *∆Np73* expression in GC is relatively weak (*r* = 0.22, *p* = 0.2), in contrast to what is observed in esophagus cancers (*r* = 0.68, *p* = 0.03). Similarly, our own analysis of the TCGA shows no correlation (*r* = −0.07) between the mRNA levels of *TP73* and *HIC1* in GC (personal data), suggesting that other mechanisms should be involved. For instance, expression of *∆Np73* can be induced by *gastric acid* via *pro-inflammatory cytokines* (TNFα, IL-1β) [388]. In fact, only a few studies have focused on the regulation of p73 expression in GC. For instance, without identifying which isoform, it was shown that p73 expression is increased by *serum starvation*, inhibition of *methylases* (5-Aza-DC), and the *extracellular matrix* protein Periostin in GC cell lines [332,389].

The effect of methylase inhibitors might be explained by the methylation of p73 promoter in *EBV* infected GC [390,391]. EBV impacts also on *TP73* at the protein level. For instance, the viral protein EBNA3C directly interferes with p73 in the nucleus to stabilize *ΔNp73* [292]. In addition, elevated expression of *∆Np73* is caused by an increased binding of p73 on *∆Np73 promoter* consecutive to the displacement by the EBV protein LMP-1 of the repressive polycomb 2 complex component *EZH2* and the induction of epigenetic changes by *JNK-1*. As these protein-protein interactions have been described in other types of cancer, they remain to be confirmed in GC cells.

The expression and activity of p73 is also affected by *H. pylori*, contributing to the response of the gastric epithelium to the infection. For instance, *H. pylori* infected tissues show a strong p73 staining [392]. *In vitro*, the infection leads to induction of *TAp73 protein stability* (but decreases the mRNA levels) and activation of various target genes such as *PUMA*, *NOXA* and *FASR* in GC cells and primary mouse gastric epithelial cells. In parallel, the infection decreases the binding of *TAp73* to *∆Np63* due to a decrease of *∆Np63* protein and mRNA levels. The elevated expression of *TAp73* is necessary for the infection to induce cell death. The increase in p73 protein levels is progressive and peaks at 72h post-infection in AGS cells [283]. Hence, TAp73 displays anti-tumoral properties to protect the gastric epithelium from *H. pylori*-induced transformation. However, the relationship between *H. pylori* and p73 seems to be more complex, as *H. pylori* can also decrease *TAp73* transcriptional activity through the induction of the isoform *∆133p53* [291].

P73 interacts with another oncogenic pathway, the *TGF-β* pathway [393]. The transforming growth factor-β (TGF-β) is a multifunctional cytokine involved in several biological processes such as cell differentiation, proliferation, and apoptosis. In GC, mRNA level of the ligands *TGF-β1*/*2* are significantly higher [394] and *TAp73α* participates in a TGF-β-induced apoptosis [395]. At the molecular level, TGF-β increases the TAp73 protein level, the *TP73* overall mRNA level, and *TP63* to a lesser extent. Induction of TAp73 induces *BAX* and *PUMA*, which is required for TGF-β-induced apoptosis. Hence, these studies suggest that TAp73 has antitumor activity by transducing the effect of TGF-β.

It has been accepted that *TAp73* is a mediator of the response to chemotherapy in various biological systems. DNA damage induces *TAp73* activity via phosphorylation by the tyrosine kinase cAbl [396] and increases *TAp73* mRNA levels through an upregulation of E2F1 after its activation by the DNA damage inducible kinases CHK1/2 [397,398]. This was also observed in non-cancerous cells [371]. Similarly, induction of *TAp73* expression upon *cisplatin* treatment was observed in GC cells [339]. In this case, the expression of TAp73 is partly positively controlled by *HDAC4*. Interestingly, *HDAC4* expression is elevated in GC, especially in the diffuse subtype, correlates with poor prognosis, and its expression is downregulated upon treatment with cytotoxic drugs. Hence, *HDAC4* participates in a complex regulatory control of p73 expression in GC in response to chemotherapy. However, this precept has been questioned recently in GC, as Dr. Qiang showed that overexpression of *TAp73* seems to inhibit BAX and NOXA expression, to increase *Cyclin D1* expression, and confers resistance to 5-FU and doxorubicin [399,400]. In addition, the protein expression of *TAp73* appears to be downregulated in AGS cells upon 24h of treatment with these drugs. This hypothesis of a pro-survival activity of *TAp73* has also been brought forward by two additional studies suggesting that *TAp73* may play a role in GC cell metabolism. For instance, the *casein kinase 2 inhibitor CX-4945* elicits an anti-Warburg effect and inhibits gastric tumorigenesis through the downregulation of TAp73 [401]. However, this study has to be taken with precaution as some results are difficult to interpret. Nonetheless, this pro-survival function of TAp73 fits with the observation that TAp73-induced *phospho-fructokinase-1* transcription promotes the Warburg effect and cell proliferation [402]. Hence, the exact role of TAp73 in GC tumorigenesis and aggressiveness remains *debated*, highlighting the need for further and more precise investigations.

In summary, ∆Np73 seems to have an elevated expression in GC, which correlated with a poor prognosis and inhibition of p53/TAp73 activity. This is in agreement with the hypothesis that ∆Np73 has oncogenic properties in GC. However, the causes of the ∆Np73 overexpression during tumorigenesis are unclear as well as the transcriptome that is under ∆Np73 control. Therefore, there remains a lack of understanding of the exact role of TAp73 due to conflicting data. Overall, additional studies are required to fully understand the molecular events that linked TA and ∆Np73 to GC tumorigenesis and aggressiveness.

## 6. *TP63*, a Third Wheel Yet to Be Explored

*TP63* is the ancestral member from which gene duplication occurred to produce *TP73* and *TP53* [23,403]. Hence, *TP63* produces two types of N-terminus variants, based on two promoters generating *TAp63* and ∆*Np63* isoforms, and C-terminus variants due to alternative splicing (Figure 2). ΔNp63α has been described as the predominant isoform. The function of *TP63* is mostly described as to be critical for the balance between proliferation and differentiation in epithelial tissues. Besides this physiological function, mutations, or deregulated expression have been associated with different cancers. 

Tannapfel et al. were the first to investigate p63 expression using in situ hybridization and immunohistochemistry in GC [381]. They showed that p63 is expressed in about 40% of gastric tumors (*n* = 32) and is preferentially expressed in diffuse and high-grade tumors. In addition, expression was also seen in metaplasia and gastritis. These results were later confirmed showing that 48.5% of the tumors (*n* = 101) are expressing p63 [404]. As the expression of p63 in healthy stomach epithelium seems to be low [405], these observations suggested that p63 protein levels are increased upon the transformation process and tumor grade, and that it might be preferentially involved in the development of the diffuse subtype. However, it remains to identify which isoforms (TA or ∆N) are concerned by this increased expression in GC. It is of note that a partial analysis of GC cell lines with various degrees of differentiation suggested that *∆Np63* expression is higher in the poorly differentiated cell lines and overexpression of *∆Np63* induces *GATA6* expression and proliferation in MKN28 cells [403].

*∆Np63* mRNA and protein levels are strongly decreased in *H. pylori*-infected GC cells, while *TAp63* remains unchanged [392]. The decrease in *∆Np63* expression reduces its interaction with TAp73, favoring an increase in the transcriptional activity of TAp73. Concomitantly, the TAp73β protein level is increased by stability, contributing to apoptosis by the induction of *NOXA* and *PUMA*. Hence, the silencing of ∆Np63 in GC cells stimulates apoptosis [403]. The inhibition of *∆Np63* expression seems to be mediated by an increased expression of *MDM2* caused by the *H. pylori* lipopolysaccharide (LPS) via the downregulation of *DICER* and *miR-375* that targets *MDM2* [406].

*TP63* may also be involved in *EBV*-mediated transformation. Indeed, elevated expression of *∆Np63* allows a prolonged latency phase of the virus cycle by directly inhibiting the expression of *BARF1* [407]. *BARF1* is considered a viral oncogene in epithelial cells, has immune-modulating properties and is expressed as an early gene regulated by the immediate early EBV protein *R* during viral lytic replication. The repression of *BARF1* by ∆Np63 could explain why the latency phase is restricted to esophageal and GC since *∆Np63* expression is selectively elevated in those cancers, but not in lymphomas.

In summary, p63 expression is induced in GC and absent in normal gastric epithelium. This expression is specifically increased in diffuse and advanced cancers and is affected by *H. pylori* and EBV infection. Most of the existing information about the expression and role of p63 concerns ∆Np63 showing elevated expression and that it could inhibit the activity of TAp73 in vitro. However, no information exists on TAp63 or any type of physical or functional interaction with p53 or p53 mutants. In addition, there are no animal studies indicating any function of p63 in GC. Hence, additional studies are required to understand the exact function of the different isoforms of p63 in GC aggressiveness. 

## 7. Discussion and Perspective: p53 and Its Galaxy in Gastric Cancer

Since its discovery in the 90^s^, the p53 family has been intensively investigated and a vast quantity of information regarding fundamental molecular mechanisms and translational/applied observations have been published. It is clear that in various cancers *TP53* and its paralogues are playing an important role in tumorigenesis and the response to chemotherapies, and likely to other therapies as well. 

In gastric cancer, the presence of *TP53* mutations at an *early stage* and its presence *homogeneously* within the tumor indicates a key role in gastric epithelium tumorigenesis and should be considered as “*driver*” mutations. These mutations, in particular *5 hot spot* mutations (R175H, Y220C, R248Q/W, R273C/H/S, and R282W), impact on multiple levels on the patient by favoring a *hypoxic response*, *impairing the immune tumoral landscape*, increasing *metastasis*, reducing the *response to chemotherapy*, thus *impacting negatively on patient prognosis*. However, the importance of these mutations varies depending on the histological and molecular subtype of GC (Table 1). For instance, it is likely that *TP53* mutations are more important in the intestinal/CIN subgroup, as supported by mouse genetics and patients’ data. However, we should not overlook that p53 mutants are also present in other subtypes and that p53 can be inactivated by multiple different means, including viral oncogenes, amplification of inhibitors and methylation of positive regulators. The exact importance of p53 isoforms, as well as p63 and p73 isoforms, remains an open question since no mouse genetic experiments have yet supported any in vitro or patient-based observations. In particular, the exact role of TAp73 remains debated. It is clear, however, that p53 family isoforms are at the center of multiple pathways in GC, including HIF1A, Hippo/YAP, TGF-β, TNF-α and NF-kB, which can account for the profound and diverse impact of p53 mutants on the tumor characteristics. 

The data gathered over the years clearly allows a better understanding of the molecular mechanisms causing stomach tumorigenesis and gastric cancer elevated aggressiveness. However, these data have not been used for daily clinical routine in treating gastric cancer. The explanation of this relative failure to transfer to the clinic for the treatment of gastric cancer may reside in two facts. The first fact is the inherent complexity of the p53 family that contains more than 30 different isoforms, with sometimes opposite functions, and that participate in a multitude of signaling pathways. The second fact is the imperfections of the tools and techniques we are using to approach and investigate this complex family. Similarly, the access to tumor samples in sufficient number and diversity, their quality and their analysis using immunohistochemistry techniques that are antibody- and experimenter-dependent may also explain some contradictory results that impact translation to the clinic. In addition, the therapeutic strategies developed have been limited by the complexity of developing highly selective small chemicals targeting non-enzymatic proteins, the diversity of the mutations/alterations, the lack of pertinent animal models, and the length and cost of clinical trials. Finally, the use of the p53 family as biomarkers or in therapeutic approaches is also confronted by the lack of alternative therapies to chemotherapy in GC, as in several other types of cancer. 

## 8. Conclusions

Hence, a long road is still ahead of us in further understanding this complex family. This might require the development of innovative tools, such as highly selective nanobodies for p53 family isoforms, functional assays that can be applied to tissue samples or liquid biopsies, and automated-immunohistochemistry scanning coupled to artificial intelligence. It is also likely that the increasing use of pan-genomics and -proteomics approaches at the individual cellular level will contribute significantly in providing crucial information in the understanding of the functions of isoforms of the p53 family in the complex ecosystem developed by the cancer cells. In particular, dissecting how isoforms of the p53 family impact their environment via the expression of diffusible factors and membrane ligand/receptors, defining an “exteriome”, might provide clues to explain their impact on the immune landscape in the tumor ecosystem and provide strategies to improve the response to immunotherapy.

## Figures and Tables

**Table 1:** Molecular alterations in the p53 family and related pathways present in gastric cancer.

Histological Subgroup	Intestinal	Diffuse	Mix		ND
**Molecular subgroup**	CIN	GS	MSI	EBV	
**Infectious factor**	*H. pylori* (HP) (CagA; ROS; AID; CHAC1)			Epstein Barr Virus (EBNA1; EBNA3C; miR-BART5-3p; LMP1)	
***TP53* mutation**	41% missense + 23% truncating Li-Fraumeni mutations (ex. R175H, E287X; 5% penetrance)	11% missense + 2% truncating Li-Fraumeni mutations (ex. R175H, E287X; 5% penetrance)	24% missense + 9% truncating mutation frequency increase in late stage (33%)		
**TP53 elevated expression**	62% ∆133p53	48%			
**MDM2 alterations**	29% amplification or gain polymorphism SNP309 G/G	13% amplification or gain polymorphism SNP309 G/G	12% gain 6% missense or truncation	38% amplification or gain	
**MDM2 activators**	CTD2				MDMX; ENIGMA; AURKA
**MDM2 inhibitors**		FHIT			SMAD1; PICT1; UBTB1
**p53 activators**					PAX5; OSR; PKNOX2; BCL6B; TIP30; PHF2; miR-27b; miR100; MEG3 lncRNA
**p53 inhibitors**					TRIM59; CREP; HDAC4; SIRT1; EZH2; JMJD2B; miR-370; OnclncRNA-626; VCAN-AS1 lncRNA; lncRNA ZFPM2-AS; Hascirc0001546
**P53 target genes**					BTG4; DR5
***TP73***	elevated expression of TAp73 upon HP infection elevated ∆Np73			promoter methylation	1% missense mutation
***TP63***	decreased ∆Np63 in HP infected cells	Elevated p63 staining			

## Data Availability

Publicly available datasets were analyzed in this study. This data can be found here: https://www.cbioportal.org/study/summary?id=stad_tcga_pub (accessed on 31 November 2020).
