# Correction: Blanchet et al. Isoforms of the p53 Family and Gastric Cancer: A Ménage à Trois for an Unfinished Affair. *Cancers* 2021, *13*, 916

**DOI:** 10.3390/cancers18162625

**Published:** 2026-08-14

**Authors:** Anais Blanchet, Agathe Bourgmayer, Jean-Emmanuel Kurtz, Georg Mellitzer, Christian Gaiddon

**Affiliations:** 1 Laboratory “Streinth” (STress REsponse and INnovative Therapies in Cancer), Inserm UMR_S 1113, IRFAC, ITI InnoVec, Université de Strasbourg, 67200 Strasbourg, Francea.bourgmayer@icans.eu (A.B.);; 2 ICANS (Institut du Cancer Strasbourg Europe), 67200 Strasbourg, France

Several of the cited references in the original publication [1] have been retracted since publication. The changes are as follows:

The authors have deleted the following retracted references: Refs. [192,196,208,329,355]. In addition, the corresponding statements referring to these references have been removed from the main text, Figure 4, and Table 1. The reference list and all in-text citations have been renumbered accordingly.

This update does not include the referenced data, and the scientific conclusions have not been changed. This update was approved by the Academic Editor. The original publication has also been updated.
